# Cerebrovascular Reactivity Is Not Associated With Therapeutic Intensity in Adult Traumatic Brain Injury: A Validation Study

**DOI:** 10.1089/neur.2023.0011

**Published:** 2023-05-12

**Authors:** Logan Froese, Alwyn Gomez, Amanjyot Singh Sainbhi, Nuray Vakitbilir, Izzy Marquez, Fiorella Amenta, Kangyun Park, Kevin Y. Stein, Eric P. Thelin, Frederick A. Zeiler

**Affiliations:** ^1^ Biomedical Engineering, Rady Faculty of Health Sciences, University of Manitoba, Winnipeg, Manitoba, Canada.; ^2^ Section of Neurosurgery, Department of Surgery, Rady Faculty of Health Sciences, University of Manitoba, Winnipeg, Manitoba, Canada.; ^3^ Department of Human Anatomy and Cell Science, Rady Faculty of Health Sciences, University of Manitoba, Winnipeg, Manitoba, Canada.; ^4^ Undergraduate Engineering, Price Faculty of Engineering, Rady Faculty of Health Sciences, University of Manitoba, Winnipeg, Manitoba, Canada.; ^5^ Undergraduate Medical Education, Rady Faculty of Health Sciences, University of Manitoba, Winnipeg, Manitoba, Canada.; ^6^ Division of Clinical Neuroscience, Karolinska Institutet, Stockholm, Sweden.; ^7^ Department of Neurology, Karolinska University Hospital, Stockholm, Sweden.; ^8^ Division of Anaesthesia, Department of Medicine, Addenbrooke's Hospital, University of Cambridge, Cambridge, United Kingdom.

**Keywords:** cerebral autoregulation, critical care, high-frequency data assessment, pressure reactivity, therapeutic intensity level

## Abstract

Within traumatic brain injury (TBI) care, there is growing interest in pathophysiological markers as surrogates of disease severity, which may be used to improve and individualize care. Of these, assessment of cerebrovascular reactivity (CVR) has been extensively studied given that it is a consistent, independent factor associated with mortality and functional outcome. However, to date, the literature supports little-to-no impact of current guideline-supported therapeutic interventions on continuously measured CVR. Previous work in this area has suffered from a lack of validation studies, given the rarity of time-matched high-frequency cerebral physiology with serially recorded therapeutic interventions; thus, we undertook a validation study. Utilizing the Winnipeg Acute TBI database, we evaluated the association between daily treatment intensity levels, as measured through the therapeutic intensity level (TIL) scoring system, and continuous multi-modal–derived CVR measures. CVR measures included the intracranial pressure (ICP)-derived pressure reactivity index, pulse amplitude index, and RAC index (a correlation between the pulse amplitude of ICP and cerebral perfusion pressure), as well as the cerebral autoregulation measure of near-infrared spectroscopy-based cerebral oximetry index. These measures were also derived over a key threshold for each day and were compared to the daily total TIL measure. In summary, we could not observe any overall relationship between TIL and these CVR measures. This validates previous findings and represents only the second such analysis to date. This helps to confirm that CVR appears to remain independent of current therapeutic interventions and is a potential unique physiological target for critical care. Further work into the high-frequency relationship between critical care and CVR is required.

## Introduction

Given the limited improvement in traumatic brain injury (TBI) outcomes in the acute clinical setting, interest has grown in the analysis of continuous individual pathophysiological biomarkers.^1–6^ One of these markers is the assessment of cerebrovascular reactivity (CVR), which has been shown as a meaningful independent factor associated with mortality and poor functional outcome at 6 and 12 months post-injury.^1–6^

The most common continuous measure of CVR in TBI is the pressure reactivity index (PRx; a correlation coefficient between slow vasogenic waves of intracranial pressure [ICP] and mean arterial pressure [MAP]).^1,7^ Similarly, PRx has also been validated as a measure of the lower limit of autoregulation in large animal models of arterial hypotension and intracranial hypertension, which is another threshold associated with mortality and unfavorable outcomes.^8–13^ However, despite the promise of PRx and other continuous measures of cerebral autoregulation in TBI, our current understanding of the impact of intensive care unit (ICU)-based therapies on these metrics remains limited.^2,14–18^

Brain Trauma Foundation (BTF) guidelines primarily focus on ICP and cerebral perfusion pressure (CPP) targets, but these targets have only documented a marginal overall impact on altering measures of CVR.^14,18,19^ Moreover, other research has suggested that BTF-based therapies in critical care have a limited impact on the mediation of CVR.^14,16–23^ All these findings suggest that PRx could be an independent factor associated with outcome in TBI. This is echoed by recent work from the Collaborative European NeuroTrauma Effectiveness Research in TBI (CENTER-TBI) that demonstrated no association between PRx, and insult burden of PRx, with serially assessed therapeutic intensity level (TIL).^14,24^

However, previous work has mainly arisen from isolated centers within Europe, with limited validation to date.^14^ Moreover, past work has only been focused on the PRx measure of CVR, though other measures may have a slightly different overall response.^22^ The main reason for this is that data sets that combined high-frequency physiology with serially assessed therapeutic interventions are rare. Therefore, the goal of this article is to provide independent validation of the recent CENTER-TBI findings,^14^ evaluating the association between daily treatment intensity levels, as measured through the TIL total and subscores,^24,25^ and daily measures of CVR/cerebral physiology, leveraging the Winnipeg Acute TBI high-resolution database.

## Methods

### Patient population

From the prospectively maintained TBI database from the Winnipeg Acute TBI Laboratories, at the University of Manitoba, we retrospectively selected patients with archived high-frequency digital physiology (ICP and arterial blood pressure [ABP]). All patients included in this database are age ≥17 years, who have suffered moderate-to-severe TBI (Glasgow Coma Scale [GCS] <13), requiring admission to the surgical ICU for invasive ICP monitoring. Patients received treatment according to the BTF guidelines with the clinician blind to CVR.^26^

### Institutional review board ethics

Data were collected after full approval by the University of Manitoba Health Research Ethics Board (H2017:181 [Continuous Time Series Monitoring of Neurophysiology in Critically Ill Neurosurgical Patients; Approval Date: May 9, 2022], H2017:188 [Patient Demographics, Clinical Characteristics and Outcomes in Moderate/Severe Traumatic Brain Injury {TBI}: A Prospective Database Study; Approval Date: May 9, 2022], B2018:103 [Near Infrared Based Cerebrovascular Reactivity as a Means of Monitoring Cerebral Autoregulation and Predicting Outcome in Moderate/Severe Traumatic Brain Injury: A Pilot Study; Approval Date: Sept 12, 2022], and H2020:118 [Characterization of Multi-Modal Monitoring Cerebral Physiologic Derangements in Adult TBI: A CAHR-TBI Multi-Center Validation Project; Approval Date: Feb 21, 2022]) and the Health Sciences Centre Research Impact Committee; these are renewed on an annual basis, reconfirmed in 2022. Procedures were followed in accordance with the ethical standards of the responsible committee for human experimentation and with the Helsinki Declaration of 1975.

### Data collection

High-frequency ABP, ICP, and regional brain tissue oxygen saturation (rSO_2_) data were collected. ABP was obtained through arterial lines connected to pressure transducers zeroed at the level of the tragus (Baxter Healthcare Corp. CardioVascular Group, Irvine, CA).^27^ ICP was acquired by an intraparenchymal strain gauge probe (Codman ICP MicroSensor; Codman & Shurtlef Inc., Raynham, MA), placed in the frontal lobe, or by an extraventricular drain (EVD). rSO_2_ was determined with near-infrared spectroscopy (NIRS) regional oximetry of the left and right frontal lobes (Covidien INVOS 5100C; Medtronic: Dublin, Ireland).

All signals were recorded using digital data transfer or digitized by an A/D converter (DT9803/DT9804/DT9826; Data Translation, Marlboro, MA) and, where appropriate, sampled at a frequency of 100 Hz, using ICM+ software (Cambridge Enterprise Ltd., Cambridge, UK). Signal artifacts were removed using both manual and automated methods before further processing or analysis, identical to past work by our lab.^23,28–30^ EVD ICP signals had the opening artifacts and other erroneous data cleaned through manual inspection by a trained clinician.

TIL is a global summary of therapy used to control ICP and was collected by a trained clinician. It ranges from 0 to 38, with lower values indicating less care to mediated ICP, and has given classifications for type of treatment.^31^ Finally, arterial potential of hydrogen (pH), partial pressure of carbon dioxide (pCO_2_), and partial pressure of oxygen (pO_2_) were samples taken sporadically throughout the patient ICU stay, based on clinical decisions.

### Signal processing

Signal processing work was done with ICM+ or R statistical software (R Core Team [2019]; R: A language and environment for statistical computing; R Foundation for Statistical Computing, Vienna, Austria; URL https://www.R-project.org/). ABP and ICP were decimated over a 10-sec non-overlapping moving average filter to get MAP and ICP. CPP = MAP – ICP. PRx was derived as a Pearson correlation between 30 consecutive 10-sec windows of ICP and MAP, updated every minute.^32–34^ Pulse amplitude of ICP (AMP) was derived using Fourier analysis of the ICP pulse waveform.^9,35^ Pulse amplitude index (PAx) was derived as the correlation between slow waves of AMP and MAP,^9,35^ and RAC was derived as the correlation between slow waves of AMP and CPP.^36^ Cerebral oximetry index (COx) is a minimally invasive measure derived using the standard Pearson correlation between 30 consecutive 10-sec windows of MAP and rSO_2_, updated every minute to give COx_R and COx_L for the right and left side, respectively.^37^ PRx, PAx, RAC, and COx are all surrogate measures of CVR, that range from −1 to 1.^32–37^ Higher values indicate more impaired CVR, whereas values below ∼0.0–0.4 indicate intact CVR.^8,11,37,38^

Using the date and time stamp for each minute-by-minute data point, daily summaries were derived for all days after injury for each patient, producing:

1. Mean ICP, percentage of time with ICP >20 and 22 mm Hg, extracted from the BTF guidelines.^39^
2. Mean CPP, percentage of time with CPP <60 and >70 mm Hg, extracted from the BTF guidelines.^38,26^
3. Mean PRx, percentage of time with PRx >0, +0.25, and +0.35 literature-defined thresholds.^8,38^
4. Mean PAx, percentage of time with PAx >0 and +0.25 literature-defined thresholds.^8^
5. Mean RAC, percentage of time with RAC above −0.10 and −0.05 literature-defined thresholds.^8^
6. Mean COx, percentage of time with COx >0 and +0.30 (both right and left side) current literature-highlighted thresholds.^11,37,40^

### Statistical analysis

These daily physiological measures were then linked with the daily TIL measures for statistical analysis; thus, the data are “day-matched” data. The TIL intermediate scoring system is a daily measure of therapy intensity level for the control of ICP, consisting of the evaluation of current utilized therapies with a focus to target ICP and CPP.^24,25^ Next, the “day-match” physiology was shifted back 1 day and then paired with the daily TIL data (such that day 2 physiology was paired with day 1 TIL) to give “time-shifted” data. This was to assess the impact of TIL scores on the following day's CVR.

Normality of all continuous variables was assessed by the Shapiro-Wilk test. For visualization of the data, box plots were used to visualize the relationship between ICP, CPP, PRx, PAx, RAC, and COx variables across increasing TIL total and subscores.

Individual daily physiologies were compared with Kruskal-Wallis testing, using box plots to demonstrate the data. Using the total daily TIL score, each physiological variable and threshold were compared across increasing daily TIL, using the Jonckheere-Terpstra test with 100 permutations. Alpha was initially set at 0.05 for this test, and then Bonferroni's adjustment was applied to get an alpha for significance of 0.0005.

Given that all TIL subscores failed to demonstrate statistical significance in association with ICP, CPP, PRx, PAx, RAC, and COx variables, and that PRx is the most common CVR measure, only the percentage of time with PRx >0 will be reported in detail in the article. Identical results were found for all other variable relationships for both the day-matched and time-shifted data sheets and can be found in Supplementary Appendix D.

In order to highlight the association between CVR with therapeutic intensity, we derived both general linear fixed effects (GLM) and general linear mixed effects (GLMM) models for total TIL (response variable) versus: mean daily PRx, PAx, RAC, and COx and daily percentage of time with PRx, PAx, RAC, and COx above given thresholds. Patient examples were used to confirm the autocorrelation between daily physiological measures, using autocorrelation function (ACF) and partial ACF plots. Given that some models demonstrated significant lags in their ACF structure, for both the GLM and GLMM, a time-series autoregressive moving average adjustment was applied.^41–46^ Autoregressive and moving average lag components ranged from 0 to 2, given that most ACF structures trailed off after 2 days (lags).^41–47^ Both GLM and GLMM effects models were compared for superiority by the Akaike information criterion, Bayesian information criterion, and analysis of variance (ANOVA). Alpha for ANOVA testing was set at 0.0005 given a Bonferroni adjustment for 100 patients. Given that all models failed to reach significance for the relationship between total TIL and CVR metrics, these models are not reported in detail within the article and only mentioned in reference to the global findings of this study.

## Results

### Patient demographics

From this TBI database, 100 patients with high-frequency physiological signals and daily TIL scores available were included in this study, from which 4 had EVDs and 80 had NIRS monitoring. A total of 358 daily observations of TIL and daily physiology responses were used for this analysis (Supplementary Appendix A). Of these, median age was 42 years, with 84% being male. Median admission GCS was 7 (4–8) with the median calculated arterial partial pressure of CO_2_ at 37 mm Hg (34–40). Table 1 summarizes the demographic data.

**Table 1.** Demographic Data

Variable		Median (interquartile range) or no. (%)
No. of patients		100
Age (years)		42 (27–57)
Sex (male)		84 (84)
GCS		7 (4–8)
GCS Motor		4 (2–5)
GCS Eyes		1 (1–2)
GCS Verbal		1 (1–2)
Pupils		
	Bilateral reactive	62 (62)
	Unilateral reactive	22 (22)
	Bilateral unreactive	16 (16)
Hypoxia (yes)		37 (37)
Hypotension (yes)		12 (12)
Marshall CT score		
	V	49 (49)
	IV	19 (19)
	III	29 (29)
	II	3 (3)
Epidural hematoma (yes)		11 (11)
Daily total TIL		7.5 (6–9)
Arterial pH		7.43 (7.38–7.46)
Arterial pCO_2_ (mm Hg)		37 (34–40)
Arterial pO_2_ (mm Hg)		111 (89–140)

CT, computerized tomography; GCS, Glasgow Coma Score; pCO_2_, partial pressure of carbon dioxide; pH, potential of hydrogen; pO_2_, partial pressure of oxygen; TIL, therapeutic intensity level.

**FIG. 1.**
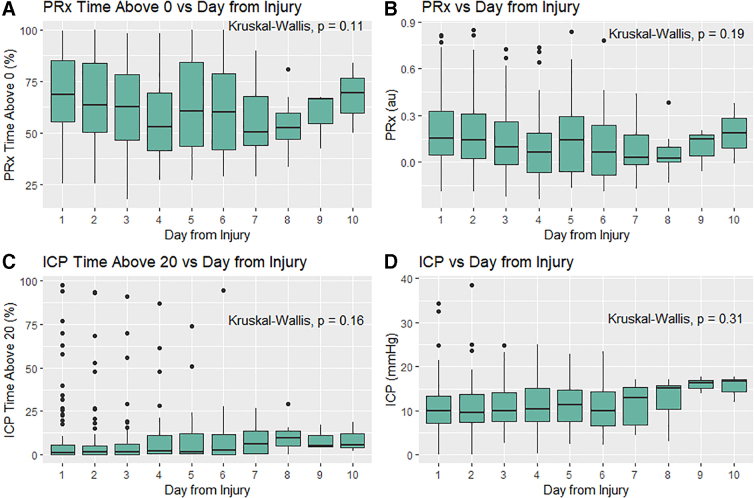
Mean daily ICP and PRx and percentage of time above key thresholds. Figure is the box plots for the mean daily ICP/PRx, mean daily percentage of time with PRx >0, and mean daily percentage of time with ICP >20 mm Hg. ICP, intracranial pressure; PRx, pressure reactivity index.

All continuous variables were found to be non-parametrically distributed. Mean daily physiological response remained relatively constant over the patient cohort, with mean daily ICP well below BTF-defined thresholds and mean daily percentage of time with PRx >0 remaining consistently >40–50%. There was no significant difference across the first 10 days on Kruskal-Wallis testing (*p* > 0.0005 for all) (Fig. 1).

### Daily total therapeutic intensity level and cerebrovascular reactivity

**FIG. 2.**
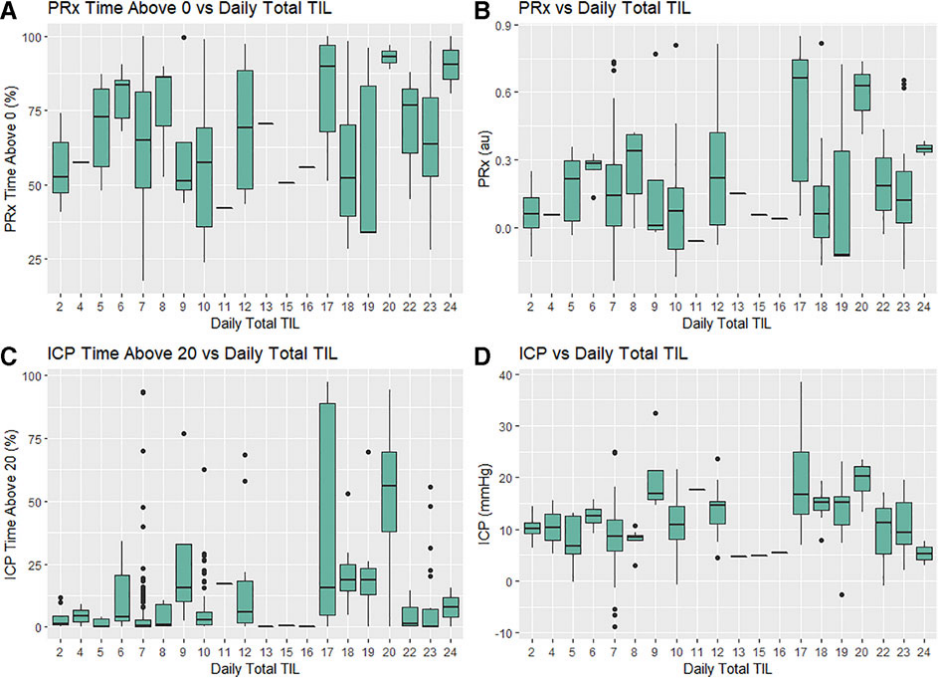
Daily total TIL versus ICP and PRx key thresholds. Figure displays the box plots of mean daily ICP/PRx, mean daily percentage of time with PRx >0, and mean daily percentage of time with ICP >20 mm Hg versus daily total TIL. ICP, intracranial pressure; PRx, pressure reactivity index; TIL, therapeutic intensity level.

Comparing total daily TIL to the daily measures of ICP and PRx (as well as CPP, PAx, RAC, and COx), the same relationships were observed for the day-matched and time-shifted data. Though increasing total daily TIL had a slight increase in daily mean ICP, percentage of time with ICP >20 mm Hg, and percentage of time with ICP >22 mm Hg, it failed to reach significance in our data and may represent abnormal physiological values coupled with care. In contrast, there was no relationship between increasing daily total TIL and daily measures of PRx or other CVR measures as mean values or percentage of time above threshold (had a *p* > 0.0005 on the Jonckheere-Terpstra test) in both the day-matched and time-shifted data sets. Supplementary Appendix B provides the box plots for the day-matched data for the other variables (Fig. 2).

### Daily therapeutic intensity-level subscores and cerebrovascular reactivity

Comparing daily TIL subscores to daily measures of PRx, identical relationships were observed for all PRx variables (i.e., mean PRx, percentage of time with PRx >0) and the other CVR variables (PAx, RAC, and COx) in both the day-matched and time-shifted data sets (Table 2). However, PAx did demonstrate some significance (even with Bonferroni testing) with the intervention of neuromuscular blockade in the day-matched data (see Supplementary Appendix C), though, apart from this, the measured subscores failed to elicit a significant response in CVR.

**Table 2.** Mann-Whitney U Testing for Percentage of Time With PRx >0 for Daily TIL Subscores for Both the Day-Matched and Time-Shifted Data

TIL subscore		Day-matched data		Mann-Whitney U* p *value	Time-shifted data		Mann-Whitney U* p *value
		Median (IQR) daily % time with PRx >0			Median (IQR) daily % time with PRx >0		
		Intervention	No intervention		Intervention	No intervention	
Fluid (vasopressors)	No. of care days	348	10	1	255	6	1
	Distribution	63.3 (48.2-81.5)	55.5 (49.6-66.2)		60.5 (46-78.7)	48.6 (42.6-59.7)	
Hyperventilation (mild)	No. of care days	13	334	0.077	8	245	0.593
	Distribution	80.6 (66.5-95)	62.7 (48.2-81)		76.6 (61.6-86.9)	60.4 (45.5-78.3)	
Hypothermia (mild)	No. of care days	4	354	0.303	1	260	1
	Distribution	82.3 (81.5–91.2)	62.9 (48.2–80.4)		84.2 (84.2–84.2)	60.3 (45.8–77.8)	
Sedation (**h**igh)	No. of care days	161	197	0.472	134	127	1
	Distribution	60.4 (44.9–77.1)	66.2 (50.3–82.6)		60.9 (42.2–73.2)	59.4 (48.2–81.6)	
Positioning	No. of care days	358	0		261	0	
	Distribution	63.2 (48.2–80.8)	—		60.3 (45.8–78.1)	—	
Neuromuscular blockade (paralysis)	No. of care days	45	313	0.994	35	226	1
	Distribution	69 (46.9–89.5)	62.5 (48.3–78.9)		64.8 (42.9–83.9)	60.1 (46.7–77.5)	
CSF drainage	No. of care days	10	348	1	8	253	1
	Distribution	55.6 (33.9–93.1)	63.2 (48.4–80.7)		37.4 (33.8–78.2)	60.4 (46.8–78)	
Hyperosmolar **t**herapy	No. of care days	177	181	0.221	147	114	1
	Distribution	60.4 (43.4–77.1)	66.4 (51.9–82.6)		60.4 (41.6–72.7)	60 (48.4–82.3)	

Table demonstrates the number of days of data and median values for percentage of time PRx >0 for all daily TIL subscore categories. For this data set, there were 3 patients who had CSF drainage over 10 days.

CSF, cerebrospinal fluid; IQR, interquartile range; PRx, pressure reactivity index; TIL, therapeutic intensity level.

## Discussion

Through the analysis of the relationship between daily total TIL and measures of CVR response, we have provided evidence that helps confirm a lack of significant impact of current critical care treatments on multi-modal monitoring-derived measures of CVR.^14^ This is only the second article and, to our knowledge, the second data set with time-matched serially assessed TIL values and high-frequency cerebral physiology.^14^ The findings here are in keeping with recent work evaluating the impact of current BTF-based therapeutic strategies on CVR in moderate/severe TBI.^14,18,48,49^ Several important findings from this validation work deserve highlighting.

First, it can be seen that a large portion of any given day is spent with impaired CVR, in keeping with other recent multi-center studies.^2,14,22,50^ Moreover, like in other past work, there was a slightly statistically significant decrease in PRx/RAC/PAx going from the first 24 h post-injury to day 4 in our cohort.^14^ This drop in PRx/RAC/PAx mostly may represent the initial spike in impaired CVR after the primary insult that improves spontaneously over the initial days of ICU care.^3,5,6,51,52^ Factors that directly increase ICP would drive CVR impairment, given that the brain can be considered a closed container. Neuroinflammatory response occurs in the hours post-TBI, cellular edema and osmolar swelling over days, and breakdown of the blood–brain barrier results from the initial injury or extreme patient states, and all lead to increase ICP (thus potentially impairing CVR).^53–55^ Each of these factors slowly improve over time in patient care and thus may account for the decrease in our determined CVR measure over the first days of care. Likewise, the extensive time that PRx/RAC/PAx/COx remained in impaired states helps to confirm that current BTF guideline-based therapeutics do not directly improve CVR.^1,48,56^

Second, therapeutic intensity analysis in either the day-matched or time-shifted data had no relationship with CVR measures, in keeping with the only other study published to date.^14^ Like past work in the field, this suggests that overall current ICU-based treatment strategies for TBI may need to adapt, so as to address the impaired nature of CVR in patients.^14^ This highlights the need to move away from the “one size fits all” approach of current guideline therapies. Potential avenues for individualized physiological targets in TBI care include optimal CPP therapy.^33,57,58^ These measures use CPP pressure to modulate CVR to achieve the most intact state within a patient (i.e., targeting the “least bad state”). Further, the results from this current study motivate further investigation into the mechanisms involved in impaired CVR after TBI. This will require the integration of, for example, protein biomarkers with genetic profiling to determine potential mechanisms that drive impaired CVR.^59^ It is through such techniques that therapies directed at prevention and treatment may be developed, potentially leading to improved outcomes in TBI.

Third, daily TIL subscores for all individual interventions failed to show any relationship with all measures of CVR (except for PAx with neuromuscular blockade). This bolsters the only other study that compared PRx to daily TIL, finding a similar lack of association.^14^ However, it must be acknowledged that our data lack a significant number of patients in certain cohorts of TIL subscores. Moreover, even small changes in vascular reactivity metrics related to current TBI therapies may carry important implications for mortality and morbidity, given that it is still unclear how much CVR improvement is required to demonstrably improve patient outcome.

Finally, patient injury pattern, injury burden, and age all have a role in individual patient response to current TBI therapies. Recent work has documented that a statistically significant positive linear correlation was found between PAx and RAC with age and no clear difference in biological sex categories.^22^ This earlier study also suggested that AMP-based CVR indices may be better positioned to detect impairment in TBI patients with advancing age. In this study, we reconfirmed that AMP-based CVR indices behave similarly to PRx, failing to be linked to daily TIL measures. Thus, it reconfirms both the similarity in these measures and limited impact from therapeutic intensity on CVR.

### Limitations/future directions

Despite the validating findings, there are several limitations of our study that require highlighting. First, the overall patient number was low, at 100. Added to the number of patients in the only other study that assessed daily TIL, this means that ∼350 patients have been assessed in this manner globally to date.^14^ Similar data need to be replicated to assess the impact of interventions on autoregulatory response. Further, the impact of TIL subgroups measures is unclear, and thus the impact on cerebrovascular measures was modest at best. Given the nature of observational retrospective studies, it is difficult to identify whether the treatment pre-mediates physiological response or *vice versa* (i.e., “the chicken or the egg” conundrum). Moreover, therapies like neuromuscular blockade and high sedation for ICP are typically only used in the most refractory elevated ICP cases; thus, larger sample sizes are needed to capture higher-frequency events/therapies.

Future studies would benefit from higher-frequency data collection for TIL, core temperature, brain temperature, sedation dosing, and vasopressor dosing, in order to determine whether any temporal response would be observed between these treatments and CVR. Past work has noted that temperature has an association with CVR measures and that it influences cerebral microcirculation.^60–64^ In patients who receive rewarming (post-hypothermia), brain temperature increased resulting in stable ICP but increased PRx values^60^ and temperature determined throughout the body resulted in a linear positive relationship to Cox.^64^ Along with this, systemic temperature has been linked to metabolic response,^61–64^ which would influence cerebral vessels,^62^ and plays in important role in cerebral autoregulation. Likewise, TIL scores and subscores are relatively gross measures of therapeutic intensity and lack high temporal resolution; thus, the momentary impact on cerebral physiology goes undocumented. As such, the results of this study should be considered preliminary and require studies to evaluate the association between vascular reactivity, ICP, CPP, and other cerebral physiological measures, using time-series physiology data with detailed treatment annotations. Such work is the ongoing focus of both our group and ongoing multi-center initiatives.^65–68^

## Conclusion

Through this study, there appears to be no association between daily measures of therapeutic intensity and multi-modal-based measures of CVR derived from ICP and NIRS. Such findings support previous preliminary findings and the need for a larger investigation of the development of therapies aimed at the prevention and treatment of CVR.

## Acknowledgments

F.A.Z. is supported through the Manitoba Public Insurance (MPI) Professorship in Neuroscience/TBI Research Endowment, NSERC, Canadian Institutes of Health Research (CIHR), the MPI Neuroscience Research Operating Fund, the Health Sciences Centre Foundation Winnipeg, the Canada Foundation for Innovation (CFI; Project No.: 38583), Research Manitoba (Grant No.: 3906 and 5429), and the University of Manitoba VPRI Research Investment Fund (RIF).

L.F. is supported through a Research Manitoba PhD Fellowship, the Brain Canada Thompkins Travel Scholarship, NSERC, and the Graduate Enhancement of Tri-Council Stipends (GETS)–University of Manitoba.

A.G. is supported through a CIHR Fellowship.

A.S.S. is supported through the University of Manitoba Graduate Fellowship (UMGF)–Biomedical Engineering, NSERC, and the Graduate Enhancement of Tri-Council Stipends (GETS)–University of Manitoba.

K.Y.S. is supported through the University of Manitoba R.G. and E.M. Graduate Fellowship (Doctoral) in Biomedical Engineering and the University of Manitoba MD/PhD program.

E.P.T. is supported through the Strategic Research Area Neuroscience (STRATNeuro), The Erling-Persson Foundation, Region Stockholm Clinical Research Appointment (#FoUI-981490), and Karolinska Institutet Research Grants (#2022-01576).

## Supplementary Material

Supplemental data

Supplemental data

Supplemental data

Supplemental data

## Abbreviations Used

ABP: arterial blood pressure
ACF: autocorrelation function
ANOVA: analysis of variance
BTF: Brain Trauma Foundation
COx: cerebral oximetry index
CPP: cerebral perfusion pressure
CVR: cerebrovascular reactivity
EVD: extraventricular drain
GCS: Glasgow Coma Scale
GLM: general linear fixed effects model
GLMM: general linear mixed effects model
ICP: intracranial pressure
ICU: intensive care unit
MAP: mean arterial pressure
NIRS: near-infrared spectroscopy
PAx: pulse amplitude index
pCO_2_: partial pressure of carbon dioxide
pH: potential of hydrogen
pO_2_: partial pressure of oxygen
PRx: pressure reactivity index
rSO_2_: regional brain tissue oxygen saturation
TBI: traumatic brain injury
TIL: therapeutic intensity level
