# Supplementary material for: Cerebrovascular Reactivity Is Not Associated With Therapeutic Intensity in Adult Traumatic Brain Injury: A Validation Study
Source: Neurotrauma Rep. 2023 May 12;4(1):307–17. doi: 10.1089/neur.2023.0011 (PMC10181802; doi:10.1089/neur.2023.0011)
Supplement: Supplemental data [file Supp_AppendixD.docx]

# Appendix D. Daily TIL Sub-Scores vs Physiological Response

*These following are the physiological variable verse the Daily TIL sub-scores. The p values are from the Mann-U test using Bonferroni adjustment, bolded indicating significance. For this dataset there were 3 patients who had CSF drainage over 10 days. COx, cerebral oximetry index; CPP, cerebral perfusion pressure; CSF, cerebrospinal fluid; ICP, intracranial pressure; PAx, pulse amplitude index; PRx, pressure reactivity index; RAC, correlation of the pulse amplitude and CPP; TIL, therapeutic intensity level*

## Table D1. Mann U testing for % time with PRx above 0.25 for daily TIL sub-scores for both the day-matched and time-shifted data

| **TIL sub-score** | | **Day-matched data** | | **Mann U p value** | **Time-shifted data** | | **Mann U p value** |
| --- | --- | --- | --- | --- | --- | --- | --- |
|  |  | **Median (IQR) daily % time with PRx > 0.25** | |  | **Median (IQR) daily % time with PRx > 0.25** | |  |
|  |  | **Intervention** | **No Intervention** |  | **Intervention** | **No Intervention** |  |
| **Fluid (vasopressors)** | *# of care days* | 348 | 10 | 1 | 255 | 6 | 1 |
|  | *Distribution* | 36.4 (23.2-58.2) | 30.3 (26.6-43) |  | 33.9 (21.7-56) | 29.4 (21.9-31.6) |  |
| **Hyperventilation (mild)** | *# of care days* | 13 | 334 | 0.15 | 8 | 245 | 0.931 |
|  | *Distribution* | 62.3 (37.5-78.2) | 36.1 (22.5-56.9) |  | 43.9 (37.7-64.5) | 33.8 (21.3-55.3) |  |
| **Hypothermia (mild)** | *# of care days* | 4 | 354 | 0.687 | 1 | 260 | 1 |
|  | *Distribution* | 62.3 (58.4-64.5) | 35.9 (23.1-56.8) |  | 58.7 (58.7-58.7) | 33.4 (21.6-54.7) |  |
| **Sedation (High)** | *# of care days* | 161 | 197 | 0.598 | 134 | 127 | 1 |
|  | *Distribution* | 33.6 (21.9-54.5) | 40.6 (24.5-58.6) |  | 33.9 (20.8-45.4) | 31.6 (21.8-58.6) |  |
| **Positioning** | *# of care days* | 358 | 0 |  | 261 | 0 |  |
|  | *Distribution* | 36.1 (23.3-57) |  |  | 33.5 (21.7-55.2) |  |  |
| **Neuromuscular blockade (paralysis)** | *# of care days* | 45 | 313 | 0.44 | 35 | 226 | 1 |
|  | *Distribution* | 43.6 (27.1-72.2) | 35.8 (22.5-56.2) |  | 36.3 (23.2-63.4) | 31.8 (21.5-54.1) |  |
| **CSF Drainage** | *# of care days* | 10 | 348 | 1 | 8 | 253 | 1 |
|  | *Distribution* | 21.4 (12.8-88.3) | 36.2 (23.6-56.9) |  | 14.6 (12.8-64.6) | 33.7 (21.9-55.2) |  |
| **Hyperosmolar Therapy** | *# of care days* | 177 | 181 | 0.279 | 147 | 114 | 0.749 |
|  | *Distribution* | 33.5 (21.4-55.2) | 40.8 (26.3-58.4) |  | 33.4 (18.5-45.2) | 35.2 (22.5-59.1) |  |

*Table demonstrates the number of days of data and median values for % time PRx above 0.25 for all daily TIL sub score categories. Bold p values indicate significance for the Mann-U Whitney Test. CSF, cerebrospinal fluid; IQR, interquartile range; PRx, pressure reactivity index; TIL, therapeutic intensity level;*

## Table D2. Mann U testing for % time with PRx above 0.35 for daily TIL sub-scores for both the day-matched and time-shifted data

| **TIL sub-score** | | **Day-matched data** | | **Mann U p value** | **Time-shifted data** | | **Mann U p value** |
| --- | --- | --- | --- | --- | --- | --- | --- |
|  |  | **Median (IQR) daily % time with PRx > 0.35** | |  | **Median (IQR) daily % time with PRx > 0.35** | |  |
|  |  | **Intervention** | **No Intervention** |  | **Intervention** | **No Intervention** |  |
| **Fluid (vasopressors)** | *# of care days* | 348 | 10 | 1 | 255 | 6 | 1 |
|  | *Distribution* | 27.1 (16.4-45.3) | 22.4 (18.3-31.8) |  | 25.3 (14.3-43.1) | 22.1 (15.2-24.5) |  |
| **Hyperventilation (mild)** | *# of care days* | 13 | 334 | 0.358 | 8 | 245 | 1 |
|  | *Distribution* | 35 (26.1-72.9) | 27.1 (15.8-45.1) |  | 33.3 (26.5-44.4) | 25.3 (14.3-43.2) |  |
| **Hypothermia (mild)** | *# of care days* | 4 | 354 | 1 | 1 | 260 | 1 |
|  | *Distribution* | 40.7 (37-47.9) | 26.9 (16.3-45.1) |  | 44.9 (44.9-44.9) | 25.1 (14.2-42.4) |  |
| **Sedation (High)** | *# of care days* | 161 | 197 | 0.641 | 134 | 127 | 1 |
|  | *Distribution* | 25 (15.2-41.2) | 30.8 (17.7-46.1) |  | 25.3 (13.8-33.9) | 23.8 (14.9-45.4) |  |
| **Positioning** | *# of care days* | 358 | 0 |  | 261 | 0 |  |
|  | *Distribution* | 27 (16.4-45.1) |  |  | 25.2 (14.2-42.8) |  |  |
| **Neuromuscular blockade (paralysis)** | *# of care days* | 45 | 313 | 0.531 | 35 | 226 | 1 |
|  | *Distribution* | 30.6 (19.6-64.3) | 26.6 (15.8-44.6) |  | 27.5 (17.1-47.5) | 24.5 (14.2-42.3) |  |
| **CSF Drainage** | *# of care days* | 10 | 348 | 1 | 8 | 253 | 1 |
|  | *Distribution* | 12.5 (7.45-84.2) | 27.1 (17-45.1) |  | 8.27 (7.53-58.1) | 25.3 (14.5-42.6) |  |
| **Hyperosmolar Therapy** | *# of care days* | 177 | 181 | 0.302 | 147 | 114 | 0.564 |
|  | *Distribution* | 24.9 (14-42.3) | 31 (18.3-46) |  | 25 (12-33.7) | 27 (16.7-47.7) |  |

*Table demonstrates the number of days of data and median values for % time PRx above 0.35 for all daily TIL sub score categories. Bold p values indicate significance for the Mann-U Whitney Test. CSF, cerebrospinal fluid; IQR, interquartile range; PRx, pressure reactivity index; TIL, therapeutic intensity level;*

## Table D3. Mann U testing for % time with PAx above 0 for daily TIL sub-scores for both the day-matched and time-shifted data

| **TIL sub-score** | | **Day-matched data** | | **Mann U p value** | **Time-shifted data** | | **Mann U p value** |
| --- | --- | --- | --- | --- | --- | --- | --- |
|  |  | **Median (IQR) daily % time with PAx > 0** | |  | **Median (IQR) daily % time with PAx > 0** | |  |
|  |  | **Intervention** | **No Intervention** |  | **Intervention** | **No Intervention** |  |
| **Fluid (vasopressors)** | *# of care days* | 348 | 10 | 1 | 255 | 6 | 1 |
|  | *Distribution* | 49.5 (34.4-68) | 38.6 (33.8-55.1) |  | 48.7 (32.4-67.7) | 39.3 (34.7-54.5) |  |
| **Hyperventilation (mild)** | *# of care days* | 13 | 334 | 0.00649 | 8 | 245 | 0.164 |
|  | *Distribution* | 75.1 (70.2-85.3) | 49 (34.3-67.5) |  | 74.1 (69.5-78.1) | 47.6 (32.2-67.5) |  |
| **Hypothermia (mild)** | *# of care days* | 4 | 354 | 0.239 | 1 | 260 | 1 |
|  | *Distribution* | 81.2 (72.9-90.6) | 49 (34.3-67.7) |  | 66.7 (66.7-66.7) | 47.7 (32.6-67.7) |  |
| **Sedation (High)** | *# of care days* | 161 | 197 | 1 | 134 | 127 | 1 |
|  | *Distribution* | 49.2 (34.5-67) | 49.4 (33.9-69.6) |  | 49.3 (32.2-67.1) | 43.8 (33.3-71.1) |  |
| **Positioning** | *# of care days* | 358 | 0 |  | 261 | 0 |  |
|  | *Distribution* | 49.2 (34.4-68) |  |  | 48.1 (32.8-67.7) |  |  |
| **Neuromuscular blockade (paralysis)** | *# of care days* | 45 | 313 | **2.03E-05** | 35 | 226 | 0.00262 |
|  | *Distribution* | 67.3 (50.7-82.2) | 45 (32.9-65.9) |  | 66.3 (49.1-74.4) | 43.8 (30.3-65.9) |  |
| **CSF Drainage** | *# of care days* | 10 | 348 | 1 | 8 | 253 | 1 |
|  | *Distribution* | 40.7 (40.1-75.1) | 49.3 (34.2-67.8) |  | 43.5 (40.3-62.3) | 48.5 (32.1-67.7) |  |
| **Hyperosmolar Therapy** | *# of care days* | 177 | 181 | 1 | 147 | 114 | 1 |
|  | *Distribution* | 49.1 (34.3-66.9) | 50.2 (34.4-69.9) |  | 49 (30.6-66.5) | 45 (34.5-73.8) |  |

*Table demonstrates the number of days of data and median values for % time PAx above 0 for all daily TIL sub score categories. Bold p values indicate significance for the Mann-U Whitney Test. CSF, cerebrospinal fluid; IQR, interquartile range; PAx, pulse amplitude index; TIL, therapeutic intensity level;*

## Table D4. Mann U testing for % time with PAx above 0.25 for daily TIL sub-scores for both the day-matched and time-shifted data

| **TIL sub-score** | | **Day-matched data** | | **Mann U p value** | **Time-shifted data** | | **Mann U p value** |
| --- | --- | --- | --- | --- | --- | --- | --- |
|  |  | **Median (IQR) daily % time with PAx > 0.25** | |  | **Median (IQR) daily % time with PAx > 0.25** | |  |
|  |  | **Intervention** | **No Intervention** |  | **Intervention** | **No Intervention** |  |
| **Fluid (vasopressors)** | *# of care days* | 348 | 10 | 1 | 255 | 6 | 1 |
|  | *Distribution* | 23.4 (13.1-40.1) | 15.2 (14.8-33.3) |  | 22.7 (13.1-38.8) | 17.5 (14.9-32.9) |  |
| **Hyperventilation (mild)** | *# of care days* | 13 | 334 | 0.0257 | 8 | 245 | 0.401 |
|  | *Distribution* | 43.7 (38.2-61.4) | 22.8 (12.9-39.2) |  | 40.1 (38.2-46.8) | 22.2 (13-38.3) |  |
| **Hypothermia (mild)** | *# of care days* | 4 | 354 | 0.889 | 1 | 260 | 1 |
|  | *Distribution* | 37.5 (35.6-47.8) | 22.9 (13.1-40) |  | 34.6 (34.6-34.6) | 22.2 (13.1-38.8) |  |
| **Sedation (High)** | *# of care days* | 161 | 197 | 1 | 134 | 127 | 1 |
|  | *Distribution* | 24.3 (13.4-37.9) | 23 (12.9-42) |  | 24.8 (13.4-37.4) | 21 (13.1-42.9) |  |
| **Positioning** | *# of care days* | 358 | 0 |  | 261 | 0 |  |
|  | *Distribution* | 23.1 (13.1-40) |  |  | 22.5 (13.1-38.8) |  |  |
| **Neuromuscular blockade (paralysis)** | *# of care days* | 45 | 313 | **1.08E-05** | 35 | 226 | 0.00116 |
|  | *Distribution* | 39.4 (29-57.4) | 20.7 (12.5-36.3) |  | 37.7 (24.3-47.6) | 19.9 (12.4-35.2) |  |
| **CSF Drainage** | *# of care days* | 10 | 348 | 1 | 8 | 253 | 1 |
|  | *Distribution* | 17.6 (16.8-43.7) | 23.4 (13-40) |  | 19.9 (17.5-31.8) | 22.8 (13-38.8) |  |
| **Hyperosmolar Therapy** | *# of care days* | 177 | 181 | 1 | 147 | 114 | 1 |
|  | *Distribution* | 22.3 (13.1-38) | 23.4 (13-41.9) |  | 21.6 (12.7-36.6) | 23.1 (13.5-48.1) |  |

*Table demonstrates the number of days of data and median values for % time PAx above 0.25 for all daily TIL sub score categories. Bold p values indicate significance for the Mann-U Whitney Test. CSF, cerebrospinal fluid; IQR, interquartile range; PAx, pulse amplitude index; TIL, therapeutic intensity level;*

## Table D5. Mann U testing for % time with RAC above -0.1 for daily TIL sub-scores for both the day-matched and time-shifted data

| **TIL sub-score** | | **Day-matched data** | | **Mann U p value** | **Time-shifted data** | | **Mann U p value** |
| --- | --- | --- | --- | --- | --- | --- | --- |
|  |  | **Median (IQR) daily % time with RAC > -0.1** | |  | **Median (IQR) daily % time with RAC > -0.1** | |  |
|  |  | **Intervention** | **No Intervention** |  | **Intervention** | **No Intervention** |  |
| **Fluid (vasopressors)** | *# of care days* | 348 | 10 | 1 | 255 | 6 | 1 |
|  | *Distribution* | 30.9 (15.8-51.2) | 19 (12.9-25.2) |  | 26.6 (14.8-47.9) | 15.6 (9.25-23.9) |  |
| **Hyperventilation (mild)** | *# of care days* | 13 | 334 | 0.526 | 8 | 245 | 0.98 |
|  | *Distribution* | 54.4 (27.9-67.2) | 30.8 (15.8-49.7) |  | 52 (28.1-59.8) | 26.5 (14.8-46.6) |  |
| **Hypothermia (mild)** | *# of care days* | 4 | 354 | 1 | 1 | 260 | 1 |
|  | *Distribution* | 56.4 (40.7-58) | 29.6 (15.6-50.4) |  | 49.8 (49.8-49.8) | 26.1 (14.4-46.7) |  |
| **Sedation (High)** | *# of care days* | 161 | 197 | 1 | 134 | 127 | 1 |
|  | *Distribution* | 28.4 (15.8-51.7) | 31 (15.6-49.1) |  | 28.6 (15.6-49.5) | 23.3 (13.5-42.8) |  |
| **Positioning** | *# of care days* | 358 | 0 |  | 261 | 0 |  |
|  | *Distribution* | 29.9 (15.7-50.9) |  |  | 26.1 (14.4-47.2) |  |  |
| **Neuromuscular blockade (paralysis)** | *# of care days* | 45 | 313 | 0.057 | 35 | 226 | 0.171 |
|  | *Distribution* | 47.4 (22.3-64.3) | 28.2 (15.3-47) |  | 35.4 (22.9-55.3) | 25.1 (13.9-44.7) |  |
| **CSF Drainage** | *# of care days* | 10 | 348 | 1 | 8 | 253 | 1 |
|  | *Distribution* | 34.3 (10.3-72.9) | 29.6 (15.8-50.6) |  | 10.9 (10.6-57.4) | 26.2 (15-46.8) |  |
| **Hyperosmolar Therapy** | *# of care days* | 177 | 181 | 1 | 147 | 114 | 1 |
|  | *Distribution* | 28.6 (15.6-52.1) | 31 (15.9-47.1) |  | 27.3 (15.5-48.2) | 24.8 (13.8-45.3) |  |

*Table demonstrates the number of days of data and median values for % time RAC above -0.1 for all daily TIL sub score categories. Bold p values indicate significance for the Mann-U Whitney Test. CSF, cerebrospinal fluid; IQR, interquartile range; RAC, correlation of the pulse amplitude and CPP; TIL, therapeutic intensity level;*

## Table D6. Mann U testing for % time with RAC above -0.05 for daily TIL sub-scores for both the day-matched and time-shifted data

| **TIL sub-score** | | **Day-matched data** | | **Mann U p value** | **Time-shifted data** | | **Mann U p value** |
| --- | --- | --- | --- | --- | --- | --- | --- |
|  |  | **Median (IQR) daily % time with RAC > -0.05** | |  | **Median (IQR) daily % time with RAC > -0.05** | |  |
|  |  | **Intervention** | **No Intervention** |  | **Intervention** | **No Intervention** |  |
| **Fluid (vasopressors)** | *# of care days* | 348 | 10 | 1 | 255 | 6 | 1 |
|  | *Distribution* | 26.4 (13.1-46.1) | 16.3 (10.8-23.1) |  | 23.4 (12.4-43.3) | 13.1 (7.89-22.1) |  |
| **Hyperventilation (mild)** | *# of care days* | 13 | 334 | 0.433 | 8 | 245 | 0.875 |
|  | *Distribution* | 51.7 (23.6-62.6) | 26.4 (13-45.2) |  | 49.5 (23.8-55.7) | 23.4 (12.3-42.6) |  |
| **Hypothermia (mild)** | *# of care days* | 4 | 354 | 1 | 1 | 260 | 1 |
|  | *Distribution* | 50.1 (35.5-53.1) | 25.8 (13-45.6) |  | 43.6 (43.6-43.6) | 23.1 (12.1-42.9) |  |
| **Sedation (High)** | *# of care days* | 161 | 197 | 1 | 134 | 127 | 1 |
|  | *Distribution* | 25.2 (13-46.4) | 27.4 (12.9-43.4) |  | 25.6 (12.8-44.1) | 19.8 (11.7-37.2) |  |
| **Positioning** | *# of care days* | 358 | 0 |  | 261 | 0 |  |
|  | *Distribution* | 25.8 (13-46.1) |  |  | 23.2 (12.2-43.3) |  |  |
| **Neuromuscular blockade (paralysis)** | *# of care days* | 45 | 313 | 0.0377 | 35 | 226 | 0.125 |
|  | *Distribution* | 42 (20.1-63.2) | 24.1 (12.7-43.2) |  | 31.6 (19.8-51) | 21 (12-39.6) |  |
| **CSF Drainage** | *# of care days* | 10 | 348 | 1 | 8 | 253 | 1 |
|  | *Distribution* | 28.8 (8.9-68.4) | 25.8 (13-45.8) |  | 9.4 (9.11-52.7) | 23.4 (12.5-43.3) |  |
| **Hyperosmolar Therapy** | *# of care days* | 177 | 181 | 1 | 147 | 114 | 1 |
|  | *Distribution* | 25.3 (13-46.7) | 27.4 (12.9-43.1) |  | 23.6 (12.5-43.3) | 20.4 (12-41.7) |  |

*Table demonstrates the number of days of data and median values for % time RAC above -0.05 for all daily TIL sub score categories. Bold p values indicate significance for the Mann-U Whitney Test. CSF, cerebrospinal fluid; IQR, interquartile range; RAC, correlation of the pulse amplitude and CPP; TIL, therapeutic intensity level;*

## Table D7. Mann U testing for % time with COx_L above 0 for daily TIL sub-scores for both the day-matched and time-shifted data

| **TIL sub-score** | | **Day-matched data** | | **Mann U p value** | **Time-shifted data** | | **Mann U p value** |
| --- | --- | --- | --- | --- | --- | --- | --- |
|  |  | **Median (IQR) daily % time with COx_L > 0** | |  | **Median (IQR) daily % time with COx_L  > 0** | |  |
|  |  | **Intervention** | **No Intervention** |  | **Intervention** | **No Intervention** |  |
| **Fluid (vasopressors)** | *# of care days* | 348 | 10 | 0.0489 | 255 | 6 | 0.714 |
|  | *Distribution* | 58.9 (51.6-66.3) | 74 (70.2-78.7) |  | 60.2 (53.4-66.8) | 71.4 (67.2-74) |  |
| **Hyperventilation (mild)** | *# of care days* | 13 | 334 | 0.127 | 8 | 245 | 0.169 |
|  | *Distribution* | 51.4 (44.6-54.6) | 59.2 (51.8-66.3) |  | 50.5 (47.5-52.4) | 60.5 (54.3-66.9) |  |
| **Hypothermia (mild)** | *# of care days* | 4 | 354 | 1 | 1 | 260 | 1 |
|  | *Distribution* | 58.2 (49.9-64.3) | 59.2 (51.9-66.4) |  | 59.2 (59.2-59.2) | 60.5 (53.9-67.3) |  |
| **Sedation (High)** | *# of care days* | 161 | 197 | 0.275 | 134 | 127 | 0.0868 |
|  | *Distribution* | 58.5 (51.8-64.2) | 61.2 (51.9-68.8) |  | 58.5 (51.9-64.1) | 65.3 (54.7-71.4) |  |
| **Positioning** | *# of care days* | 358 | 0 |  | 261 | 0 |  |
|  | *Distribution* | 59.2 (51.8-66.5) |  |  | 60.5 (54-67.2) |  |  |
| **Neuromuscular blockade (paralysis)** | *# of care days* | 45 | 313 | 0.235 | 35 | 226 | 0.0639 |
|  | *Distribution* | 53.7 (50.5-62.9) | 59.9 (52.6-66.6) |  | 55.9 (49.9-62.8) | 61.3 (54.8-67.7) |  |
| **CSF Drainage** | *# of care days* | 10 | 348 | 0.102 | 8 | 253 | 0.0511 |
|  | *Distribution* | 51.1 (49.2-53.7) | 59.6 (52.2-66.5) |  | 49.2 (47.6-53.8) | 60.9 (54.5-67.5) |  |
| **Hyperosmolar Therapy** | *# of care days* | 177 | 181 | 0.357 | 147 | 114 | 1 |
|  | *Distribution* | 58.5 (50.7-64.7) | 61 (53-67.7) |  | 58.5 (51.5-64.2) | 65.4 (56.9-70.8) |  |

*Table demonstrates the number of days of data and median values for % time COx_L above 0 for all daily TIL sub score categories. Bold p values indicate significance for the Mann-U Whitney Test. CSF, cerebrospinal fluid; IQR, interquartile range; COx_L, cerebral oximetry index left side; TIL, therapeutic intensity level;*

## Table D8. Mann U testing for % time with COx_L above 0.3 for daily TIL sub-scores for both the day-matched and time-shifted data

| **TIL sub-score** | | **Day-matched data** | | **Mann U p value** | **Time-shifted data** | | **Mann U p value** |
| --- | --- | --- | --- | --- | --- | --- | --- |
|  |  | **Median (IQR) daily % time with COx_L  > 0.3** | |  | **Median (IQR) daily % time with COx_L  > 0.3** | |  |
|  |  | **Intervention** | **No Intervention** |  | **Intervention** | **No Intervention** |  |
| **Fluid (vasopressors)** | *# of care days* | 348 | 10 | 0.0421 | 255 | 6 | 0.464 |
|  | *Distribution* | 24.8 (17.9-30.4) | 37.5 (34-41.1) |  | 25.4 (18.1-31.7) | 35.9 (32.2-38) |  |
| **Hyperventilation (mild)** | *# of care days* | 13 | 334 | 0.0341 | 8 | 245 | 0.227 |
|  | *Distribution* | 13.7 (13.2-16.8) | 24.9 (18.2-30.6) |  | 15.2 (13.4-18.2) | 25.7 (18.2-32) |  |
| **Hypothermia (mild)** | *# of care days* | 4 | 354 | 1 | 1 | 260 | 1 |
|  | *Distribution* | 13.1 (8.4-20.6) | 24.9 (18.1-31.4) |  | 31.6 (31.6-31.6) | 25.4 (18.1-32.2) |  |
| **Sedation (High)** | *# of care days* | 161 | 197 | 0.0036 | 134 | 127 | 0.000627 |
|  | *Distribution* | 22.8 (17.1-28.1) | 27 (19.7-33.8) |  | 23.1 (16.8-28) | 28.1 (22.8-35.2) |  |
| **Positioning** | *# of care days* | 358 | 0 |  | 261 | 0 |  |
|  | *Distribution* | 24.9 (18-31.2) |  |  | 25.5 (18.2-32.1) |  |  |
| **Neuromuscular blockade (paralysis)** | *# of care days* | 45 | 313 | 0.0135 | 35 | 226 | 0.00222 |
|  | *Distribution* | 17.8 (14.4-26.7) | 25.1 (19.1-32.1) |  | 18.6 (14.4-24.8) | 26.5 (19.5-33.7) |  |
| **CSF Drainage** | *# of care days* | 10 | 348 | 0.00677 | 8 | 253 | 0.0125 |
|  | *Distribution* | 15.1 (12.9-16.2) | 25 (18.5-31.5) |  | 14.5 (13.6-15.5) | 26.2 (19.3-32.6) |  |
| **Hyperosmolar Therapy** | *# of care days* | 177 | 181 | 0.0143 | 147 | 114 | 1 |
|  | *Distribution* | 23.2 (17.3-28.1) | 26.9 (19.9-33.8) |  | 23.5 (17.3-28.1) | 28.8 (22.7-35.4) |  |

*Table demonstrates the number of days of data and median values for % time COx_L above 0.3 for all daily TIL sub score categories. Bold p values indicate significance for the Mann-U Whitney Test. CSF, cerebrospinal fluid; IQR, interquartile range; COx_L, cerebral oximetry index left side; TIL, therapeutic intensity level;*

## Table D9. Mann U testing for % time with COx_R above 0 for daily TIL sub-scores for both the day-matched and time-shifted data

| **TIL sub-score** | | **Day-matched data** | | **Mann U p value** | **Time-shifted data** | | **Mann U p value** |
| --- | --- | --- | --- | --- | --- | --- | --- |
|  |  | **Median (IQR) daily % time with COx_R > 0** | |  | **Median (IQR) daily % time with COx_R  > 0** | |  |
|  |  | **Intervention** | **No Intervention** |  | **Intervention** | **No Intervention** |  |
| **Fluid (vasopressors)** | *# of care days* | 348 | 10 | 0.00987 | 255 | 6 | 0.174 |
|  | *Distribution* | 57.6 (48.9-68.3) | 82 (72.7-85) |  | 58.8 (49.9-68.9) | 76.2 (68.9-82) |  |
| **Hyperventilation (mild)** | *# of care days* | 13 | 334 | 0.0634 | 8 | 245 | 0.125 |
|  | *Distribution* | 47.1 (46.6-47.1) | 57.9 (49.3-68.5) |  | 46.6 (39.7-46.9) | 59.3 (50.1-69.1) |  |
| **Hypothermia (mild)** | *# of care days* | 4 | 354 | 1 | 1 | 260 | 1 |
|  | *Distribution* | 47.1 (36.5-54.5) | 58 (49.1-68.7) |  | 64.5 (64.5-64.5) | 59.3 (49.9-69.3) |  |
| **Sedation (High)** | *# of care days* | 161 | 197 | 1 | 134 | 127 | 1 |
|  | *Distribution* | 57.6 (49.6-67.9) | 58.6 (47.5-70.9) |  | 59 (49.9-68.1) | 60.2 (50.8-71) |  |
| **Positioning** | *# of care days* | 358 | 0 |  | 261 | 0 |  |
|  | *Distribution* | 58 (49-68.7) |  |  | 59.5 (50-69.3) |  |  |
| **Neuromuscular blockade (paralysis)** | *# of care days* | 45 | 313 | 0.102 | 35 | 226 | 0.0435 |
|  | *Distribution* | 50 (47.4-63.2) | 58.6 (49.9-70) |  | 50 (48-64.5) | 62 (51.2-70.3) |  |
| **CSF Drainage** | *# of care days* | 10 | 348 | 0.0896 | 8 | 253 | 0.0531 |
|  | *Distribution* | 48.6 (46.9-49.5) | 58.5 (49.4-69) |  | 48.6 (47.5-48.9) | 61.5 (51-69.9) |  |
| **Hyperosmolar Therapy** | *# of care days* | 177 | 181 | 1 | 147 | 114 | 1 |
|  | *Distribution* | 56.8 (48.8-67.9) | 58.7 (49.8-71.1) |  | 58.3 (49.6-67.9) | 62 (51.4-71.2) |  |

*Table demonstrates the number of days of data and median values for % time COx_R above 0 for all daily TIL sub score categories. Bold p values indicate significance for the Mann-U Whitney Test. CSF, cerebrospinal fluid; IQR, interquartile range; COx_R, cerebral oximetry index right side; TIL, therapeutic intensity level;*

## Table D10. Mann U testing for % time with COx_R above 0.3 for daily TIL sub-scores for both the day-matched and time-shifted data

| **TIL sub-score** | | **Day-matched data** | | **Mann U p value** | **Time-shifted data** | | **Mann U p value** |
| --- | --- | --- | --- | --- | --- | --- | --- |
|  |  | **Median (IQR) daily % time with COx_R  > 0.3** | |  | **Median (IQR) daily % time with COx_R  > 0.3** | |  |
|  |  | **Intervention** | **No Intervention** |  | **Intervention** | **No Intervention** |  |
| **Fluid (vasopressors)** | *# of care days* | 348 | 10 | 0.0116 | 255 | 6 | 0.195 |
|  | *Distribution* | 23.1 (16.1-32.6) | 44.7 (35.4-47.1) |  | 24.5 (16.5-33.1) | 40.5 (33-46.3) |  |
| **Hyperventilation (mild)** | *# of care days* | 13 | 334 | 0.0113 | 8 | 245 | 0.0826 |
|  | *Distribution* | 11.7 (11.4-11.7) | 23.6 (16.9-32.6) |  | 11.4 (10.8-11.6) | 24.8 (17-33.2) |  |
| **Hypothermia (mild)** | *# of care days* | 4 | 354 | 1 | 1 | 260 | 1 |
|  | *Distribution* | 18.5 (15-25.3) | 23.9 (16.5-33) |  | 37.1 (37.1-37.1) | 24.8 (16.6-33.3) |  |
| **Sedation (High)** | *# of care days* | 161 | 197 | 1 | 134 | 127 | 1 |
|  | *Distribution* | 23.6 (16.9-30.8) | 23.9 (15.7-34.8) |  | 23.9 (16.2-31.8) | 26.4 (17.9-35.6) |  |
| **Positioning** | *# of care days* | 358 | 0 |  | 261 | 0 |  |
|  | *Distribution* | 23.7 (16.5-32.8) |  |  | 24.8 (16.7-33.7) |  |  |
| **Neuromuscular blockade (paralysis)** | *# of care days* | 45 | 313 | 0.0399 | 35 | 226 | 0.0255 |
|  | *Distribution* | 17.3 (12.4-26.7) | 24.7 (17.5-33.2) |  | 17.4 (14.3-24.8) | 26.6 (18-34.6) |  |
| **CSF Drainage** | *# of care days* | 10 | 348 | 0.00952 | 8 | 253 | 0.0274 |
|  | *Distribution* | 14.6 (11.7-14.8) | 24.1 (17.3-33.1) |  | 14.5 (12.3-14.6) | 26.1 (17.3-34.1) |  |
| **Hyperosmolar Therapy** | *# of care days* | 177 | 181 | 0.552 | 147 | 114 | 0.552 |
|  | *Distribution* | 23 (15.9-30.8) | 24.4 (17.4-35.1) |  | 23.6 (15.8-31.6) | 26.8 (18.7-35.8) |  |

*Table demonstrates the number of days of data and median values for % time COx_R above 0.3 for all daily TIL sub score categories. Bold p values indicate significance for the Mann-U Whitney Test. CSF, cerebrospinal fluid; IQR, interquartile range; COx_R, cerebral oximetry index right side; TIL, therapeutic intensity level;*
