# Supplementary material for: Cerebrovascular Reactivity Is Not Associated With Therapeutic Intensity in Adult Traumatic Brain Injury: A Validation Study
Source: Neurotrauma Rep. 2023 May 12;4(1):307–17. doi: 10.1089/neur.2023.0011 (PMC10181802; doi:10.1089/neur.2023.0011)
Supplement: Supplemental data [file Supp_AppendixA.docx]

# Appendix A. Distribution of Daily TIL Scores


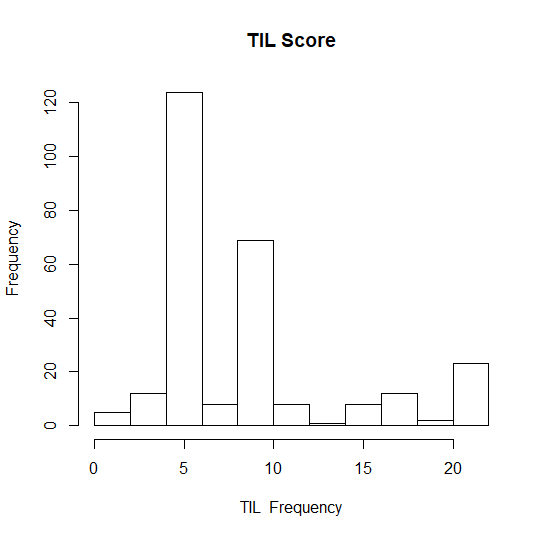


*Distribution of the Daily TIL Scores over this patient cohort. TIL, Therapeutic Intensity Level.*
