# Supplementary material for: Cerebrovascular Reactivity Is Not Associated With Therapeutic Intensity in Adult Traumatic Brain Injury: A Validation Study
Source: Neurotrauma Rep. 2023 May 12;4(1):307–17. doi: 10.1089/neur.2023.0011 (PMC10181802; doi:10.1089/neur.2023.0011)
Supplement: Supplemental data [file Supp_AppendixC.docx]

# Appendix C. Daily TIL Scores vs Physiological Response

*These following boxplots are the physiological variable verse the Daily TIL scores. The p values are the Jonckheere-Terpstra test. COx, cerebral oximetry index; CPP, cerebral perfusion pressure; ICP, intracranial pressure; PAx, pulse amplitude index; PRx, pressure reactivity index; RAC, correlation of the pulse amplitude and CPP; TIL, therapeutic intensity level.*

*
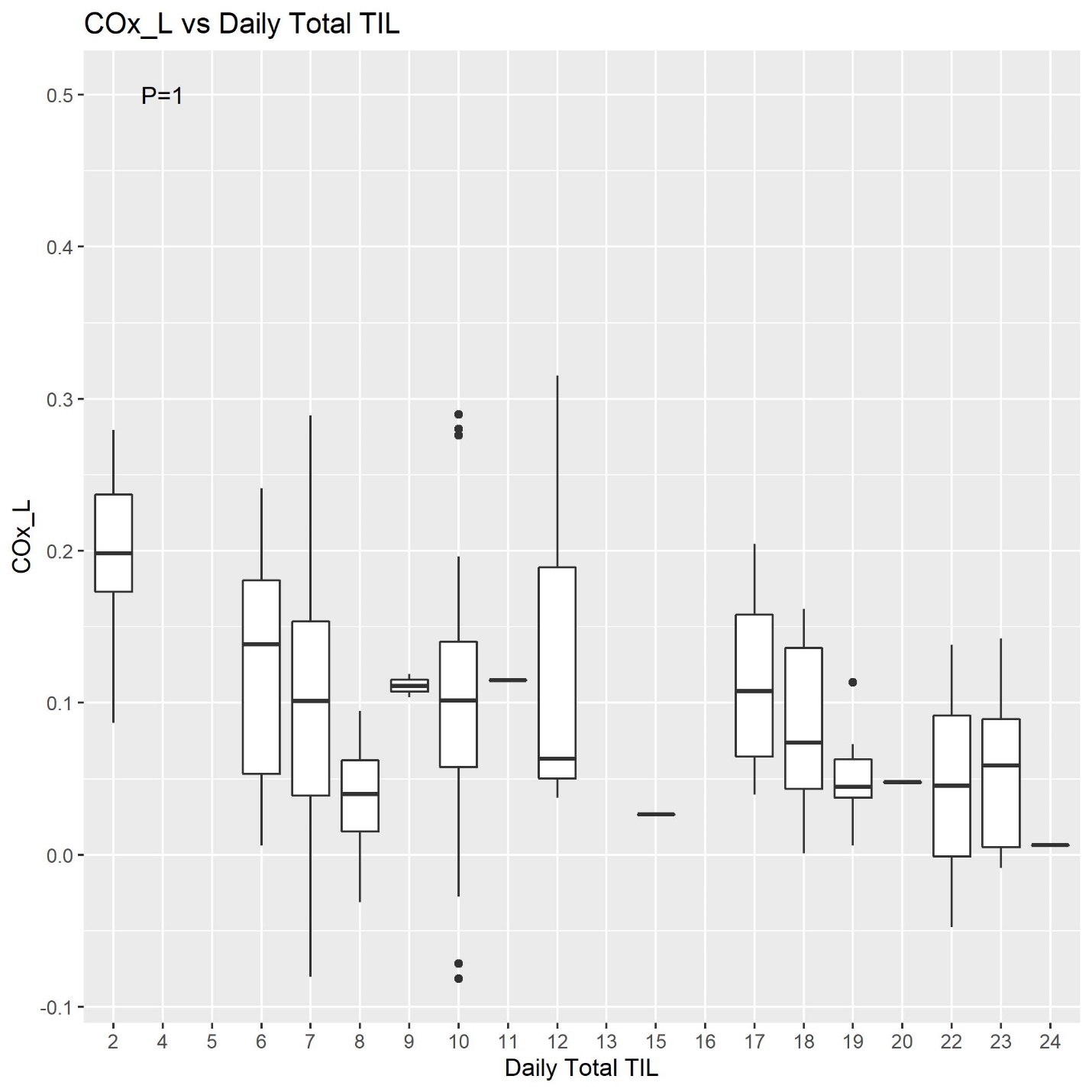
*

*
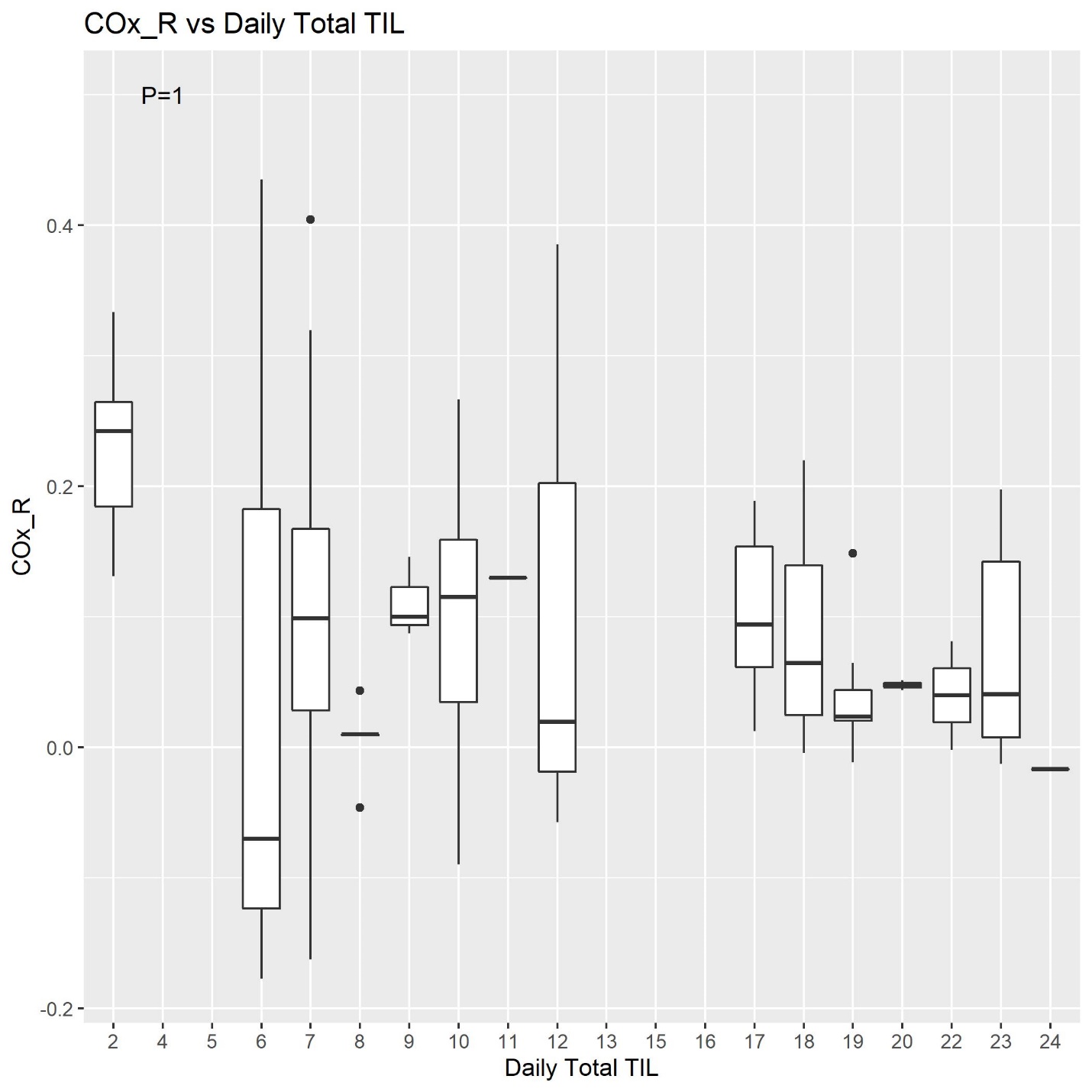

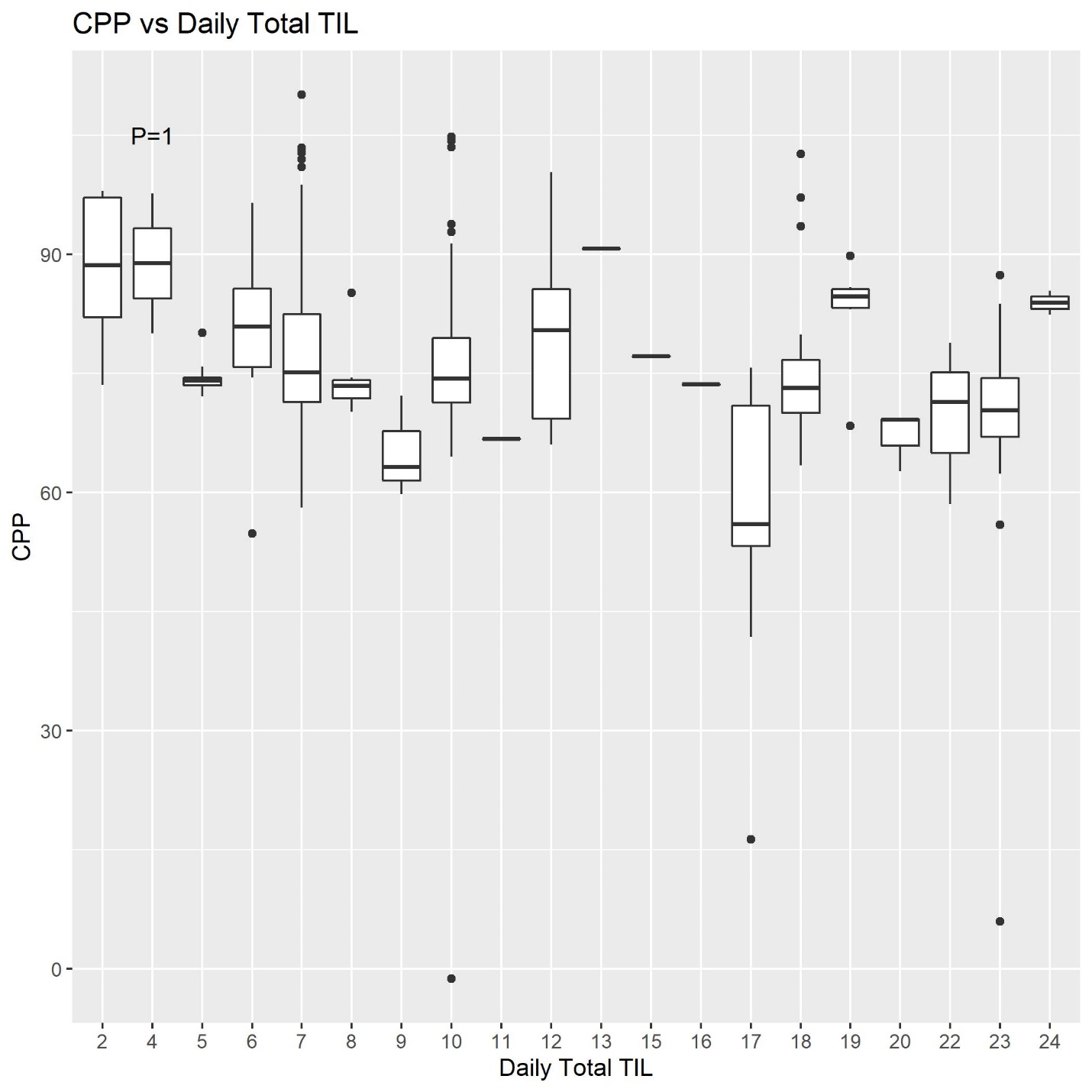

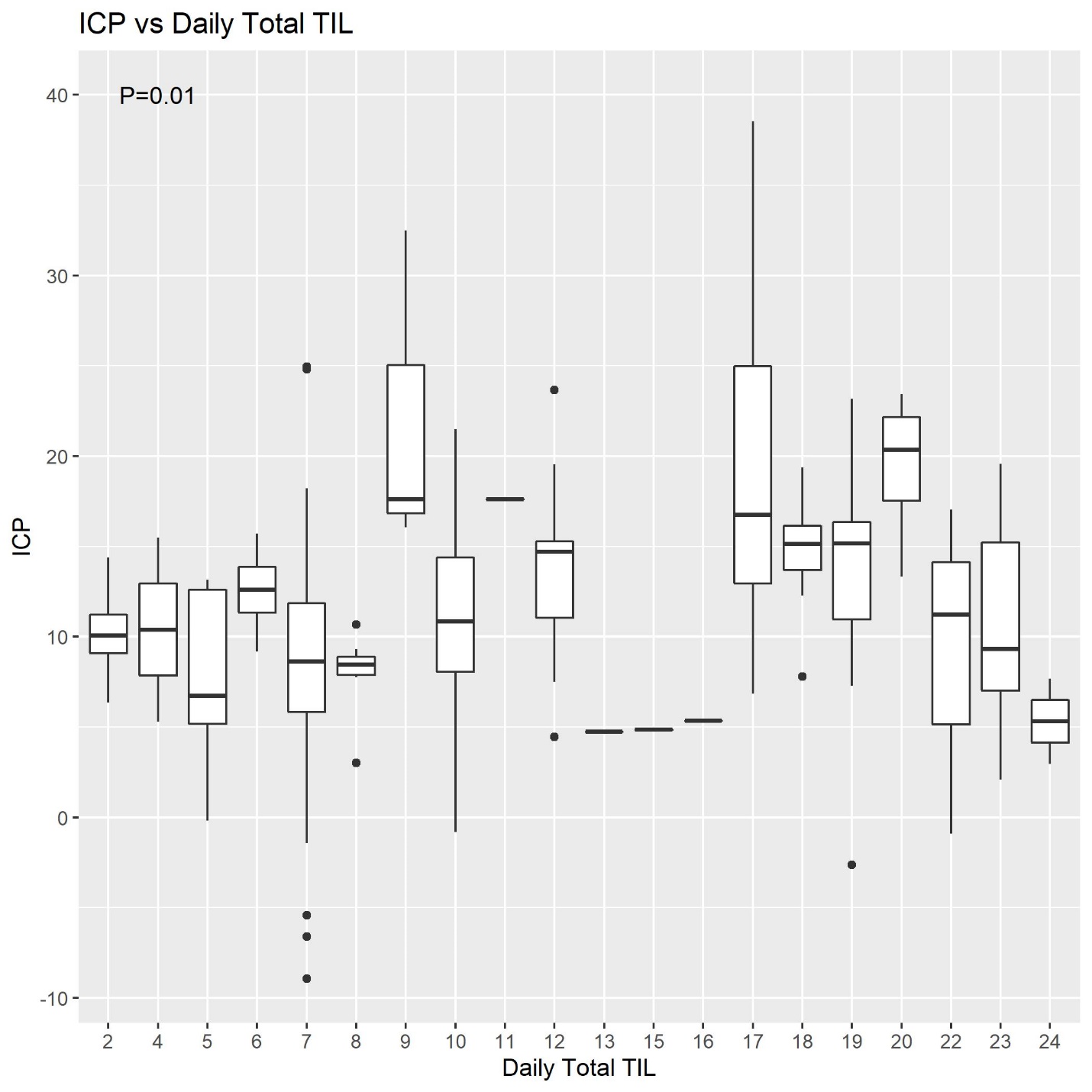

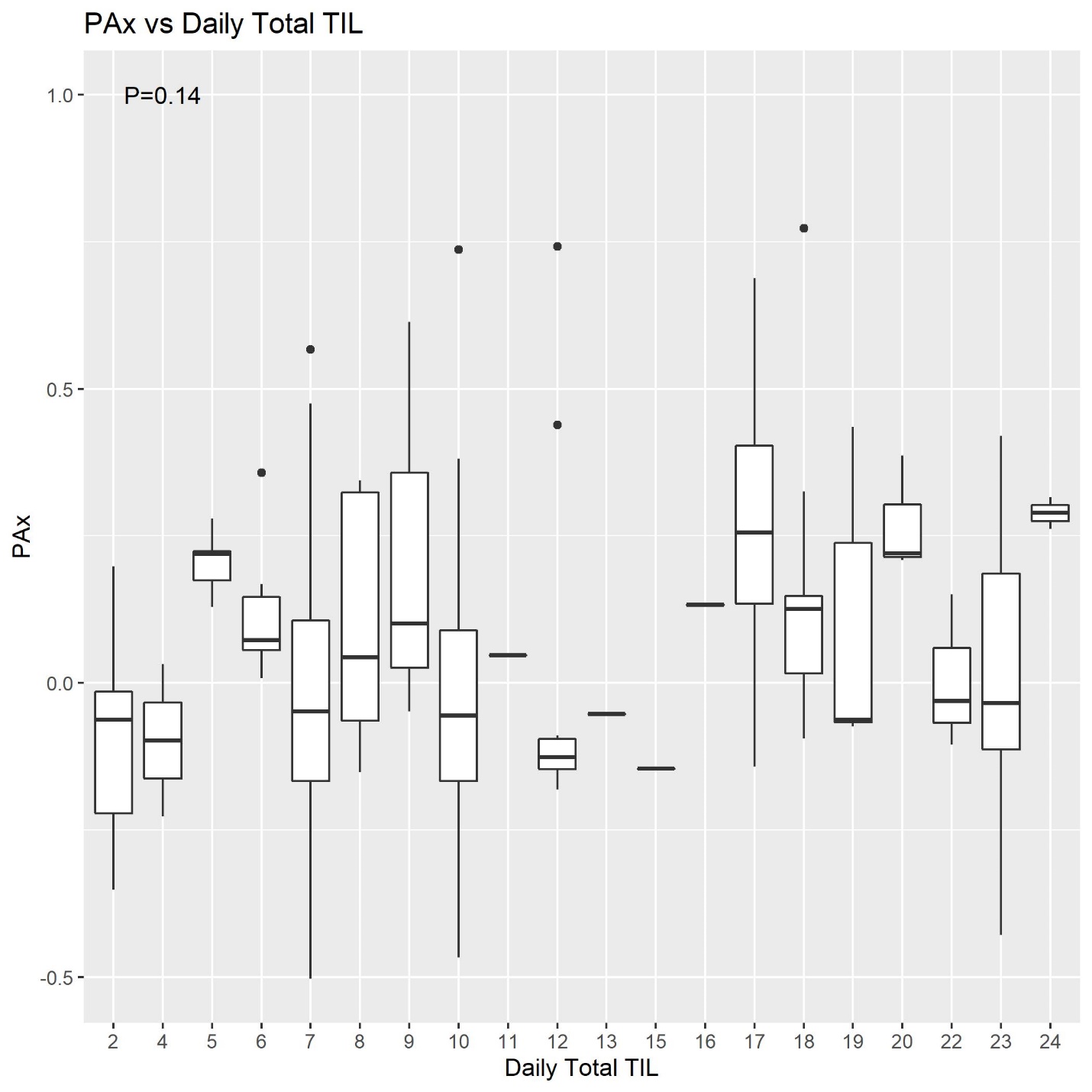

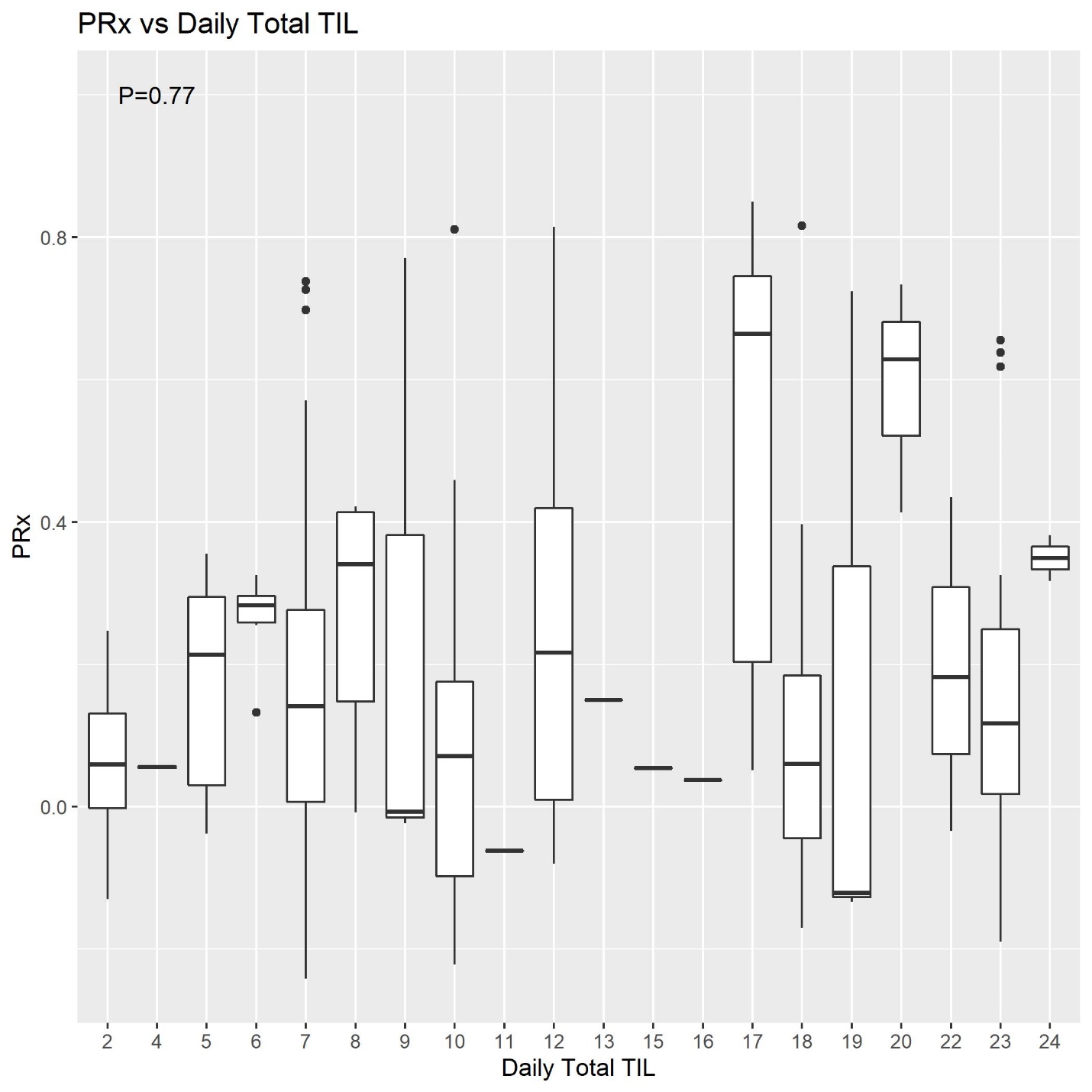

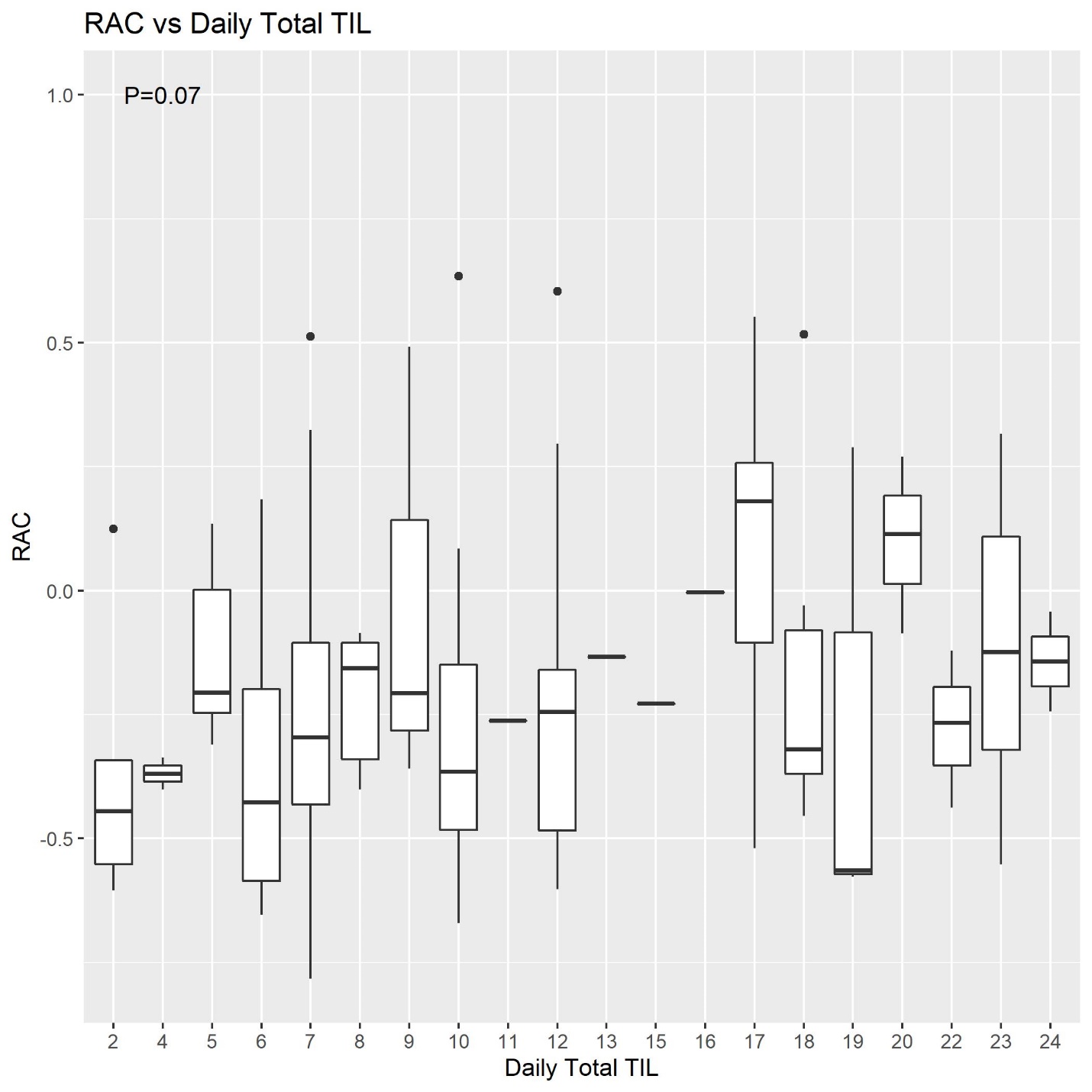

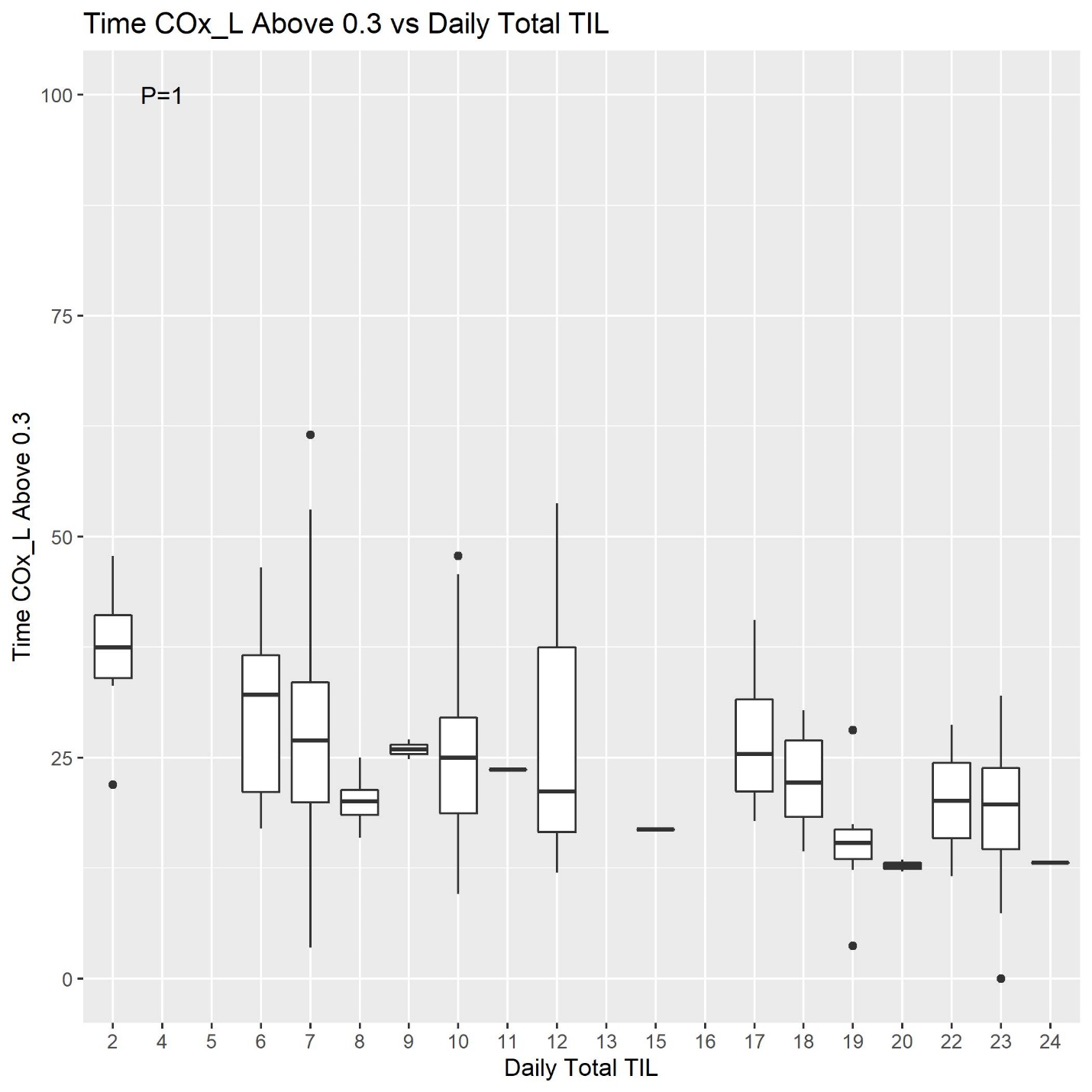

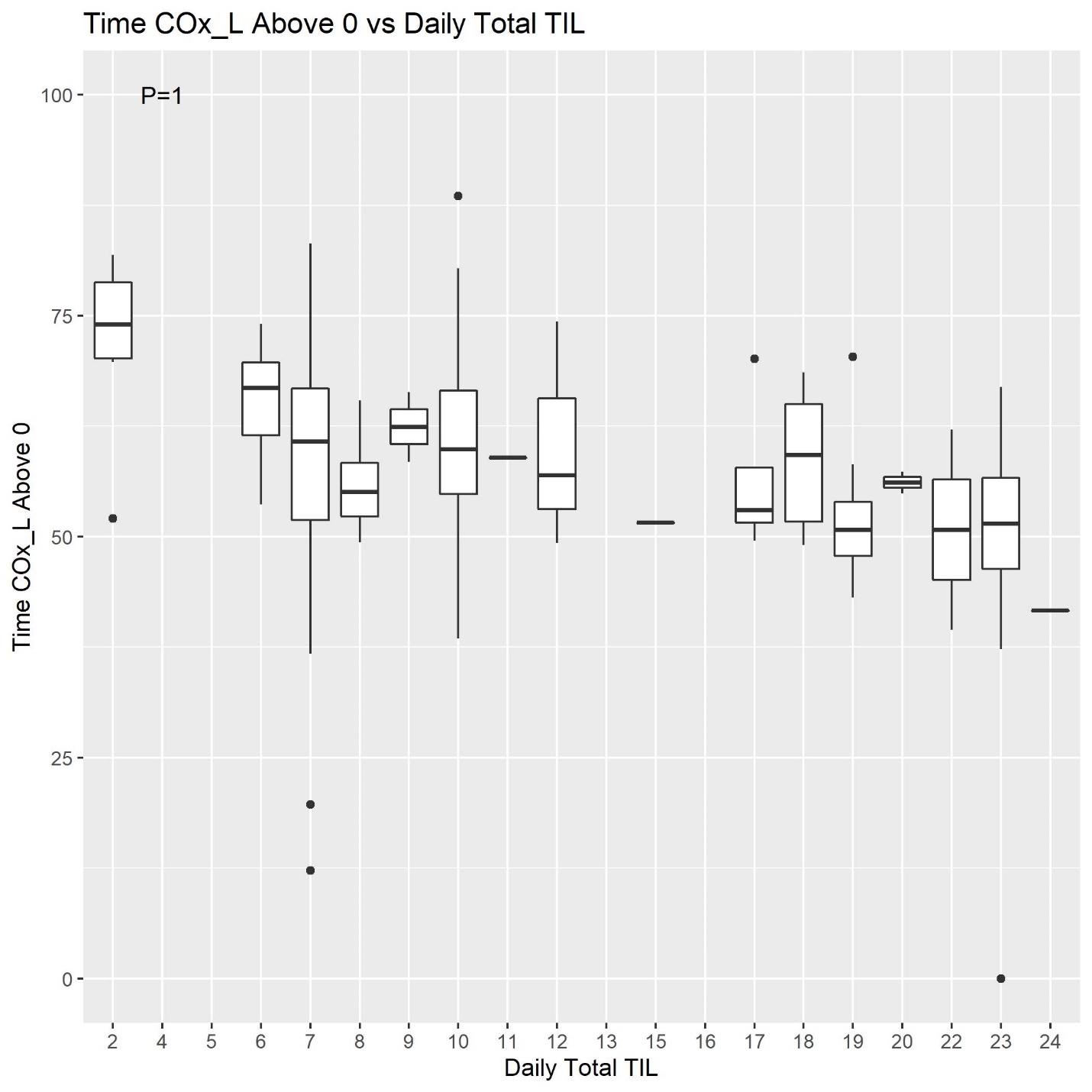

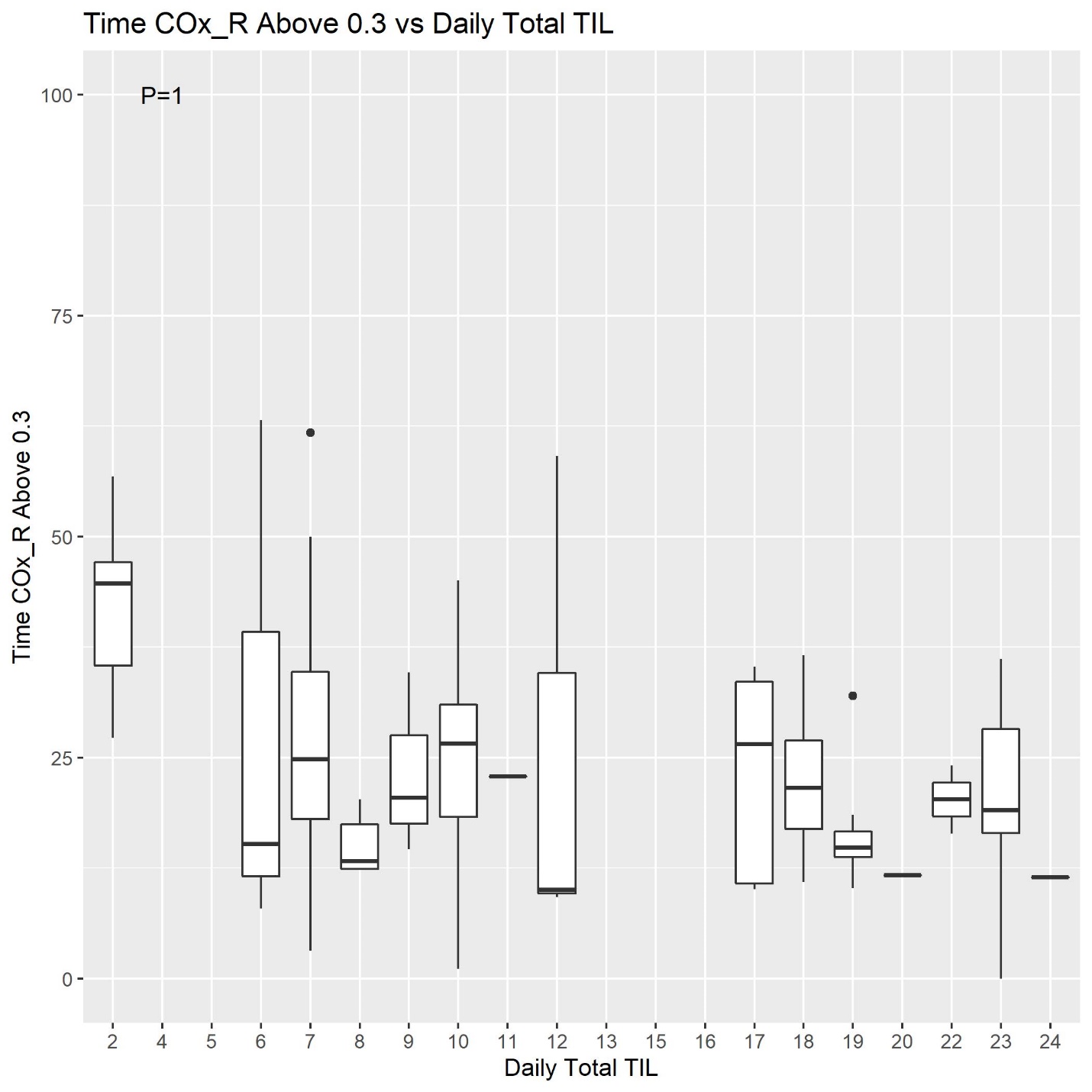

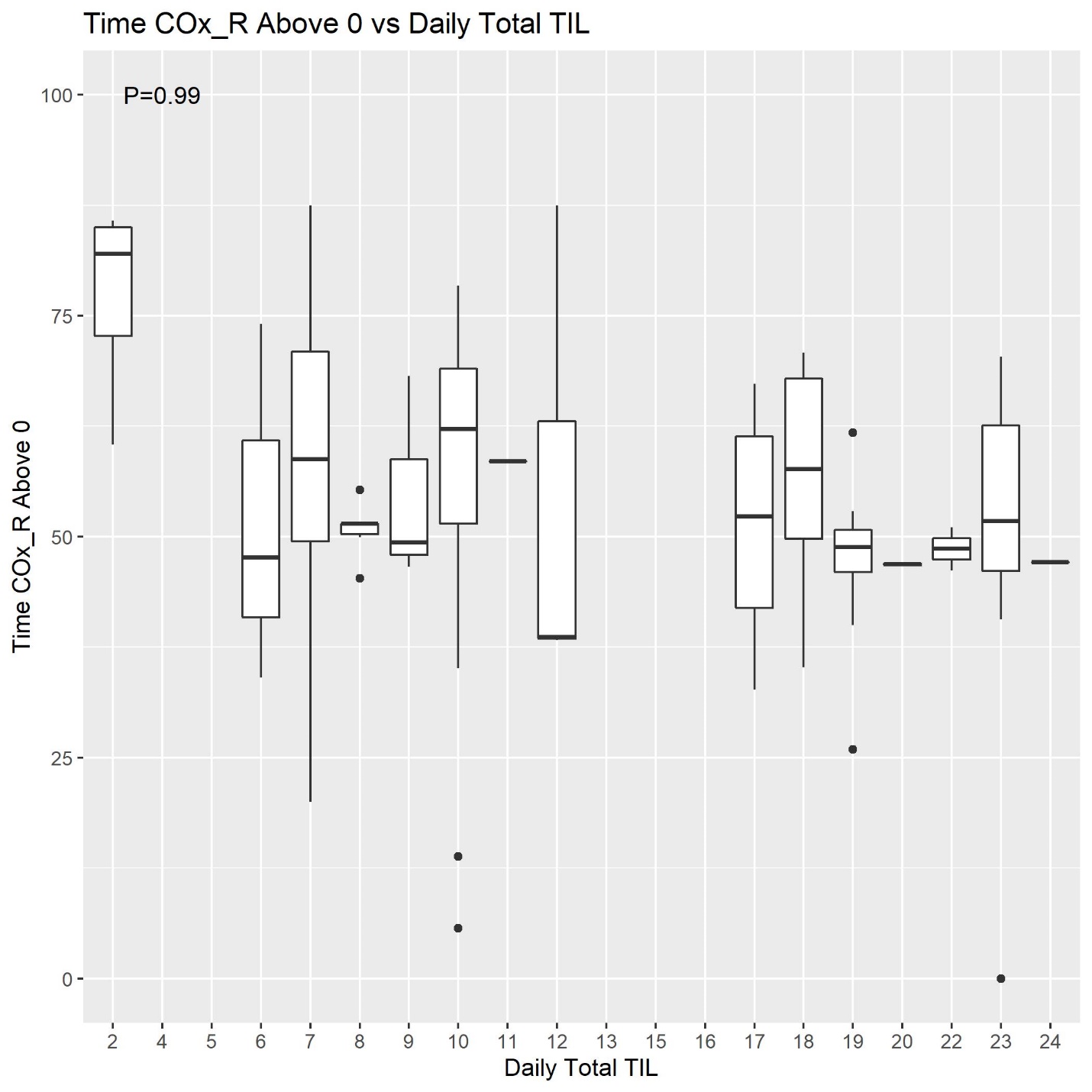

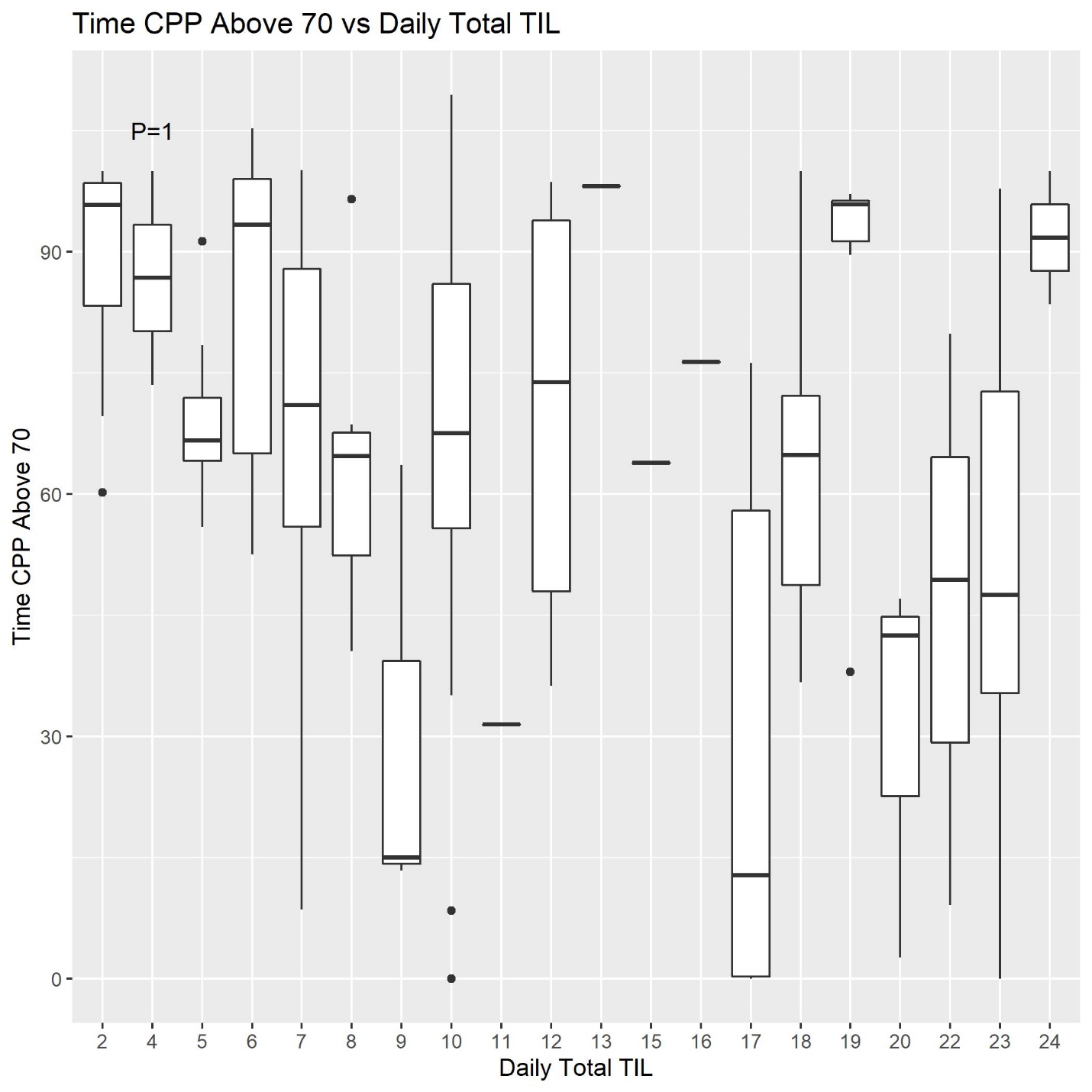

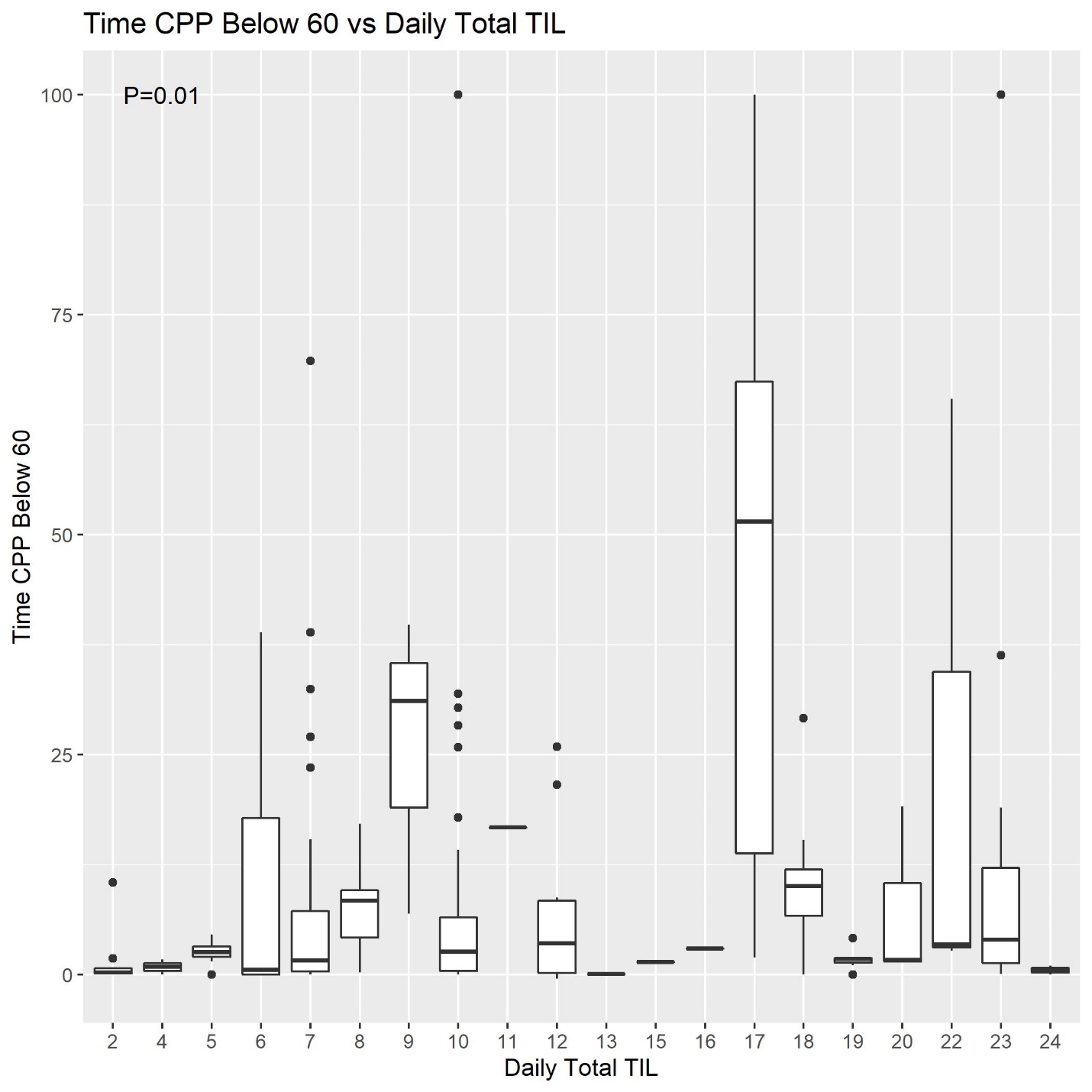

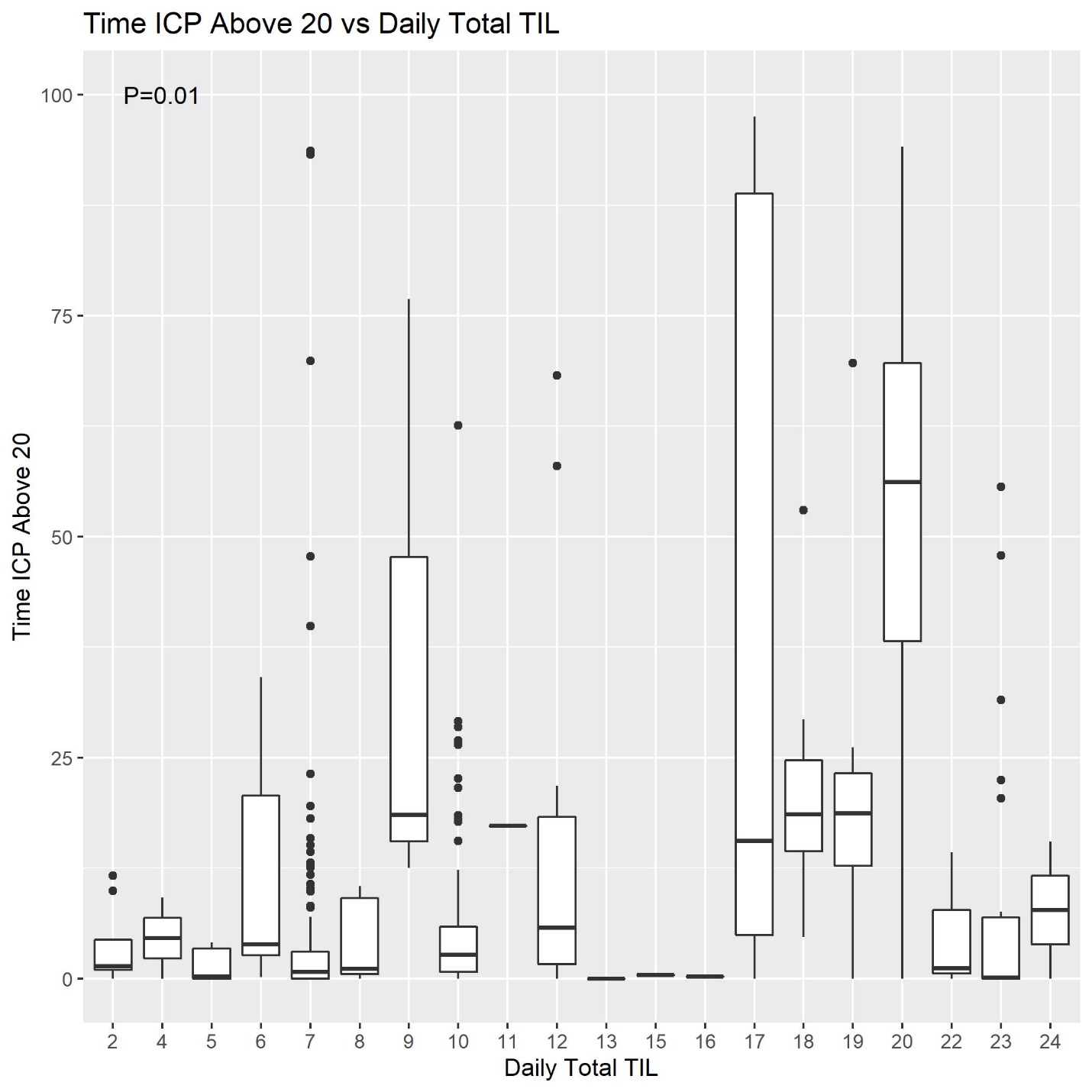

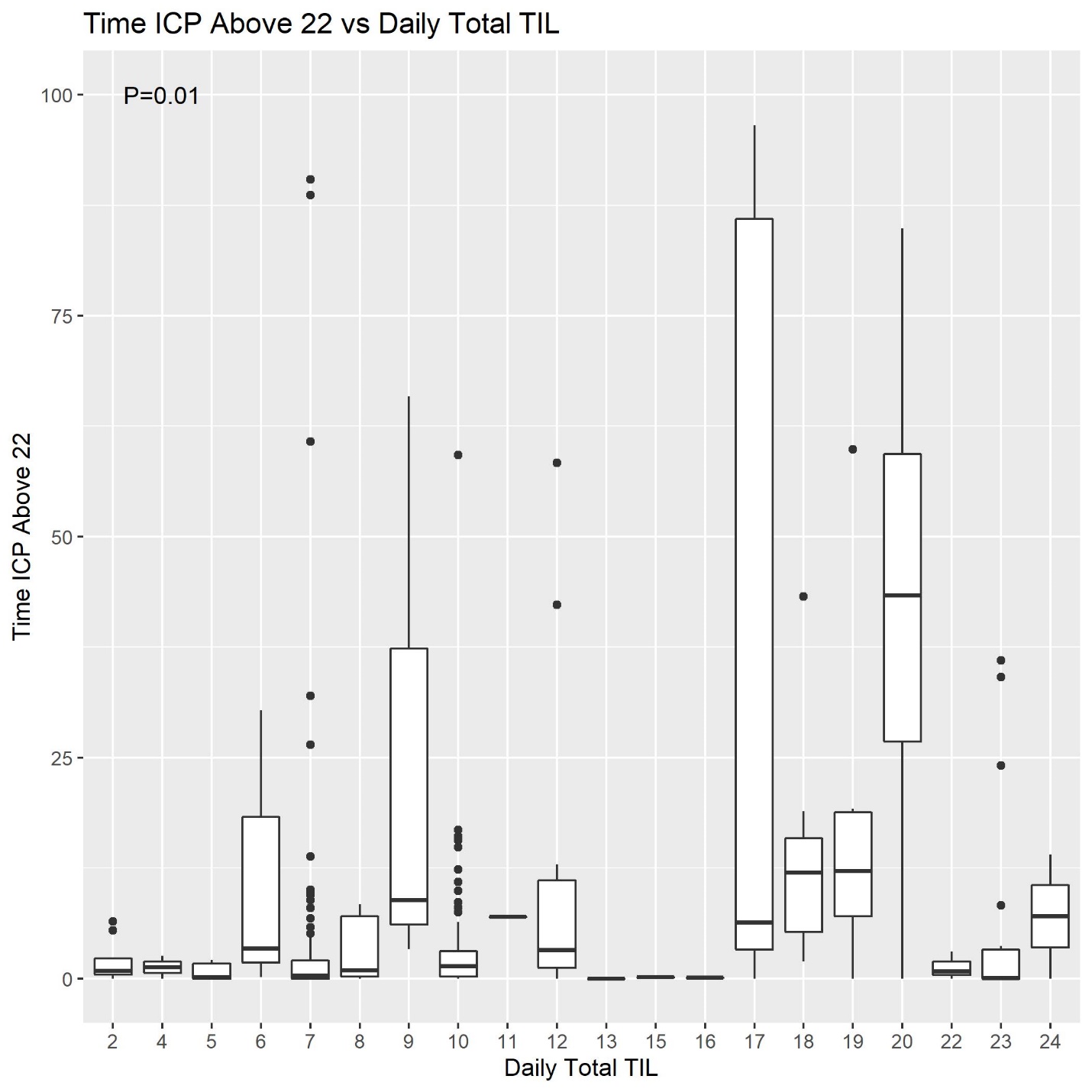

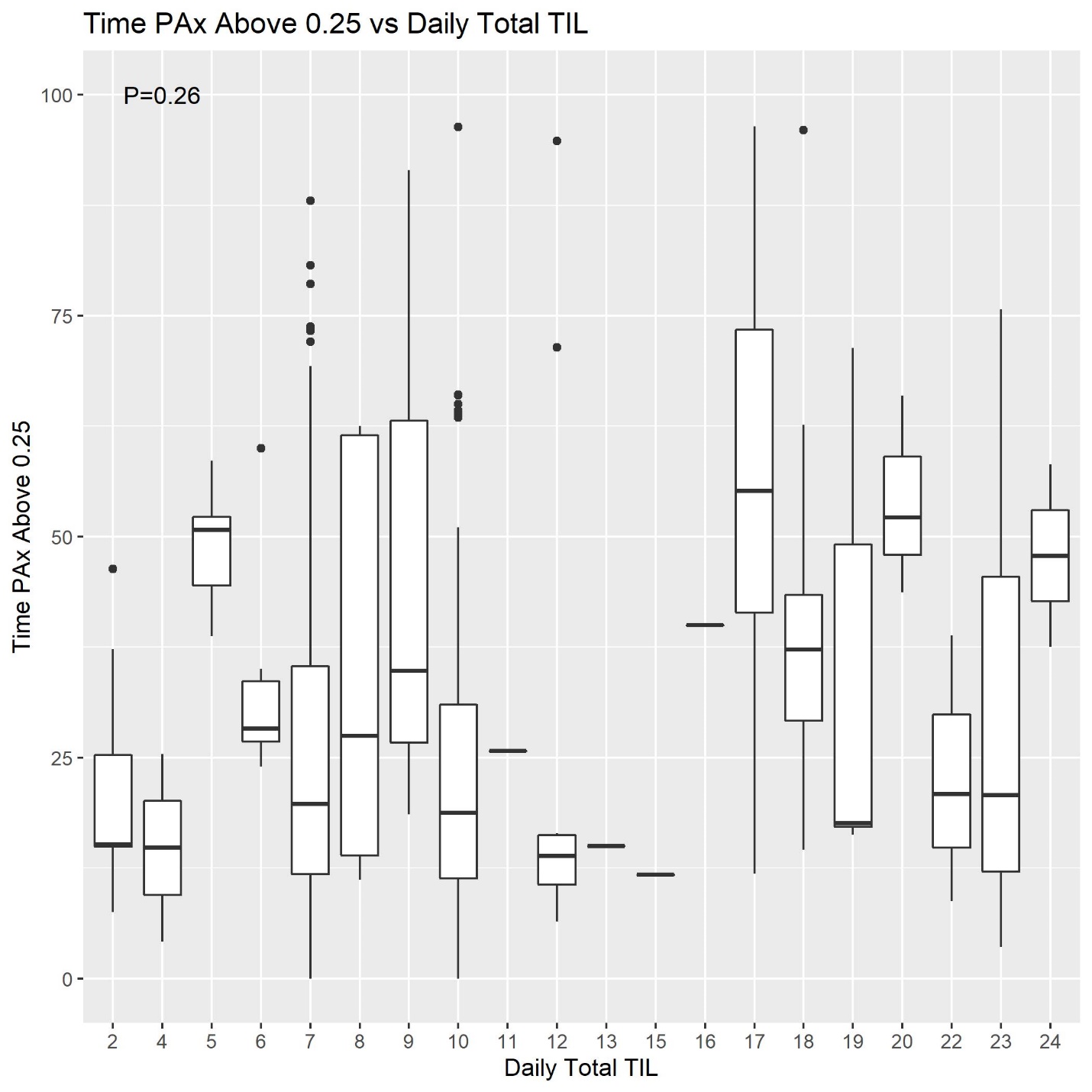

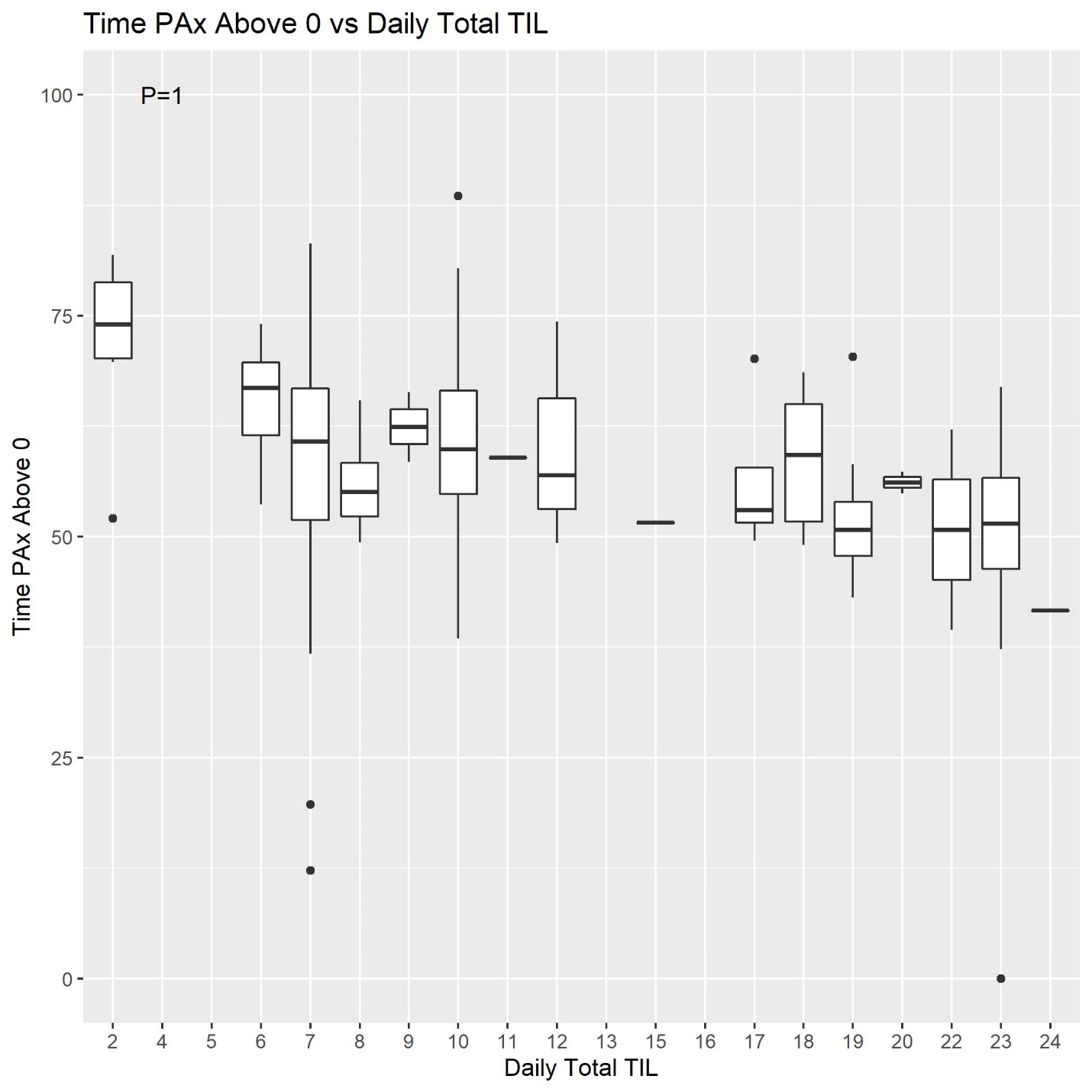

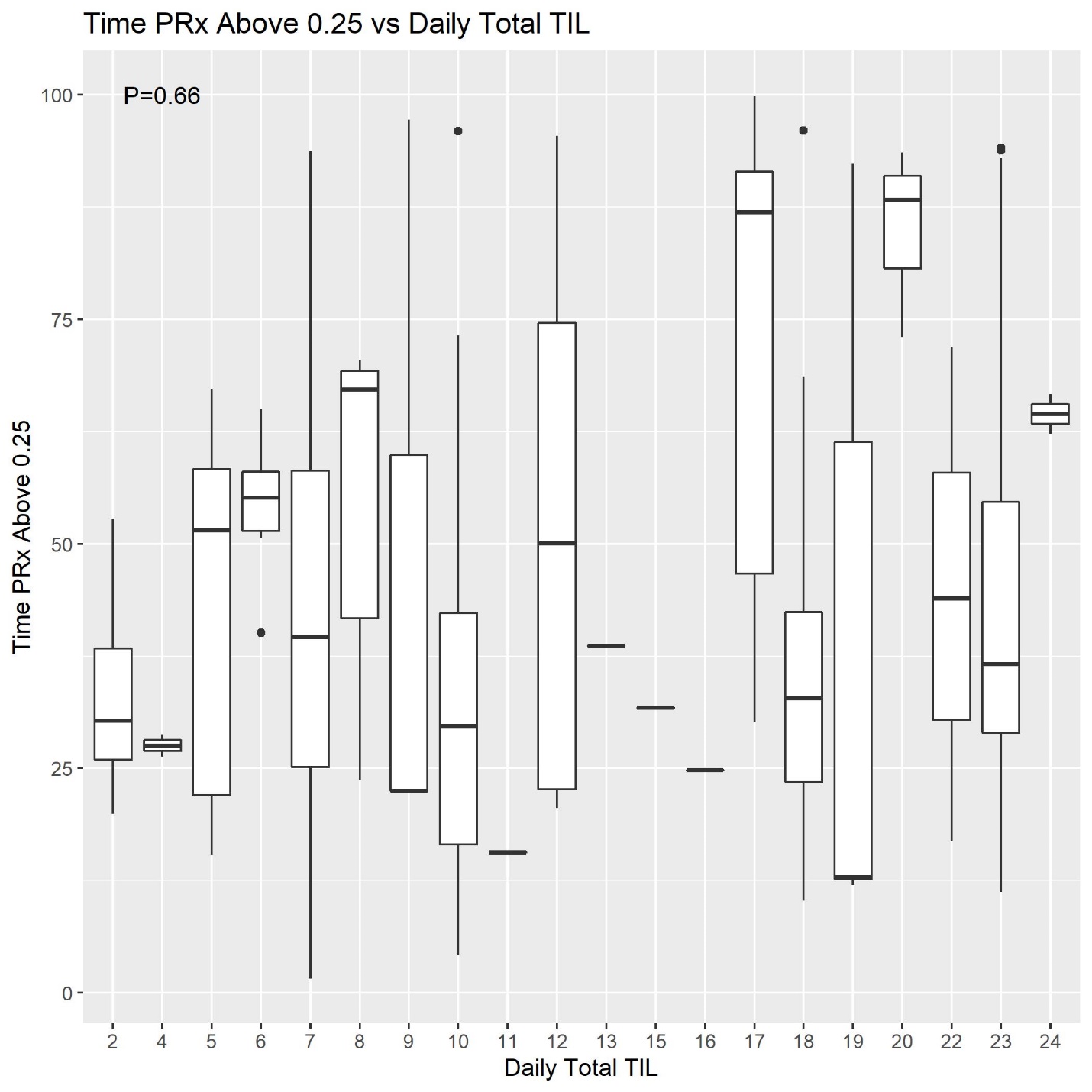

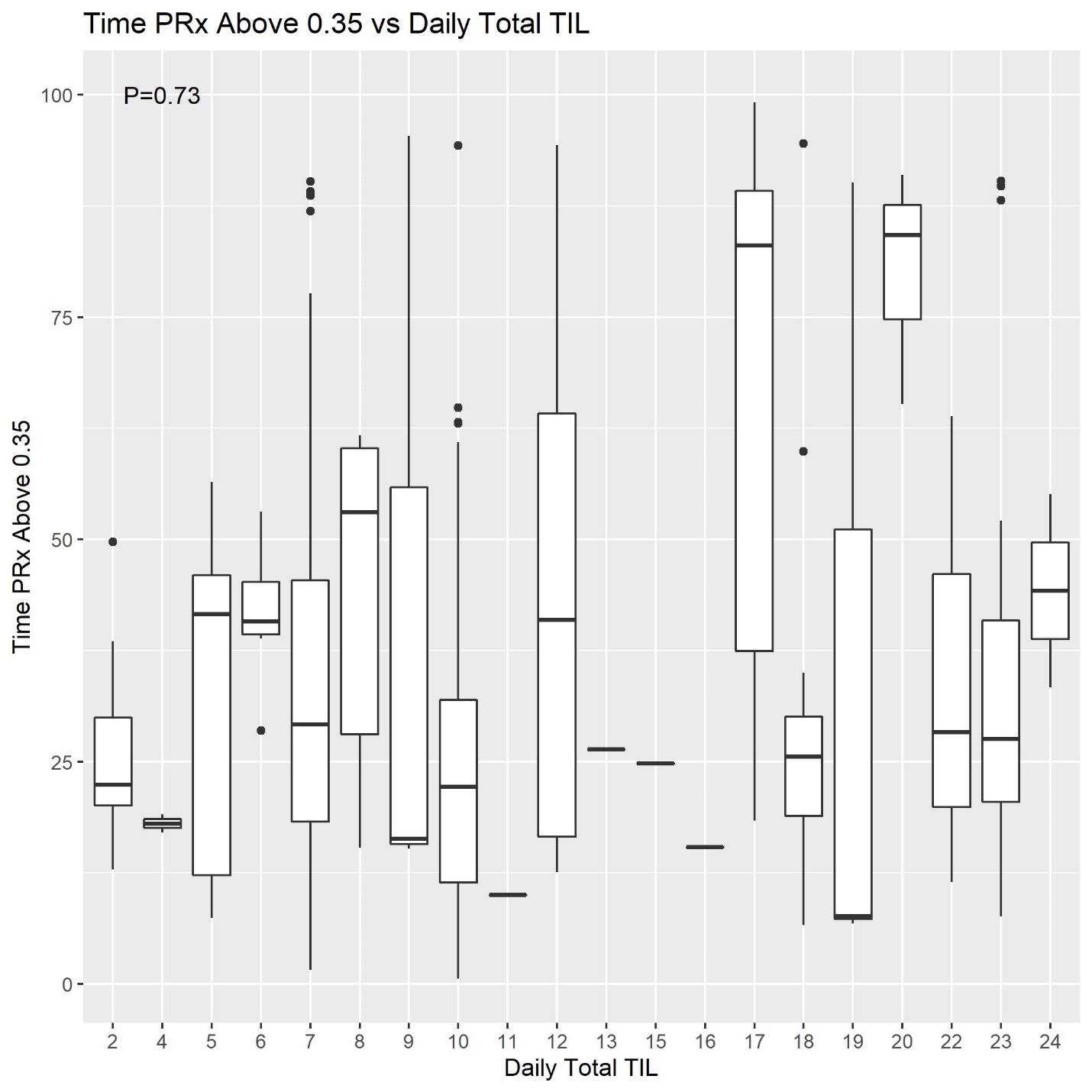

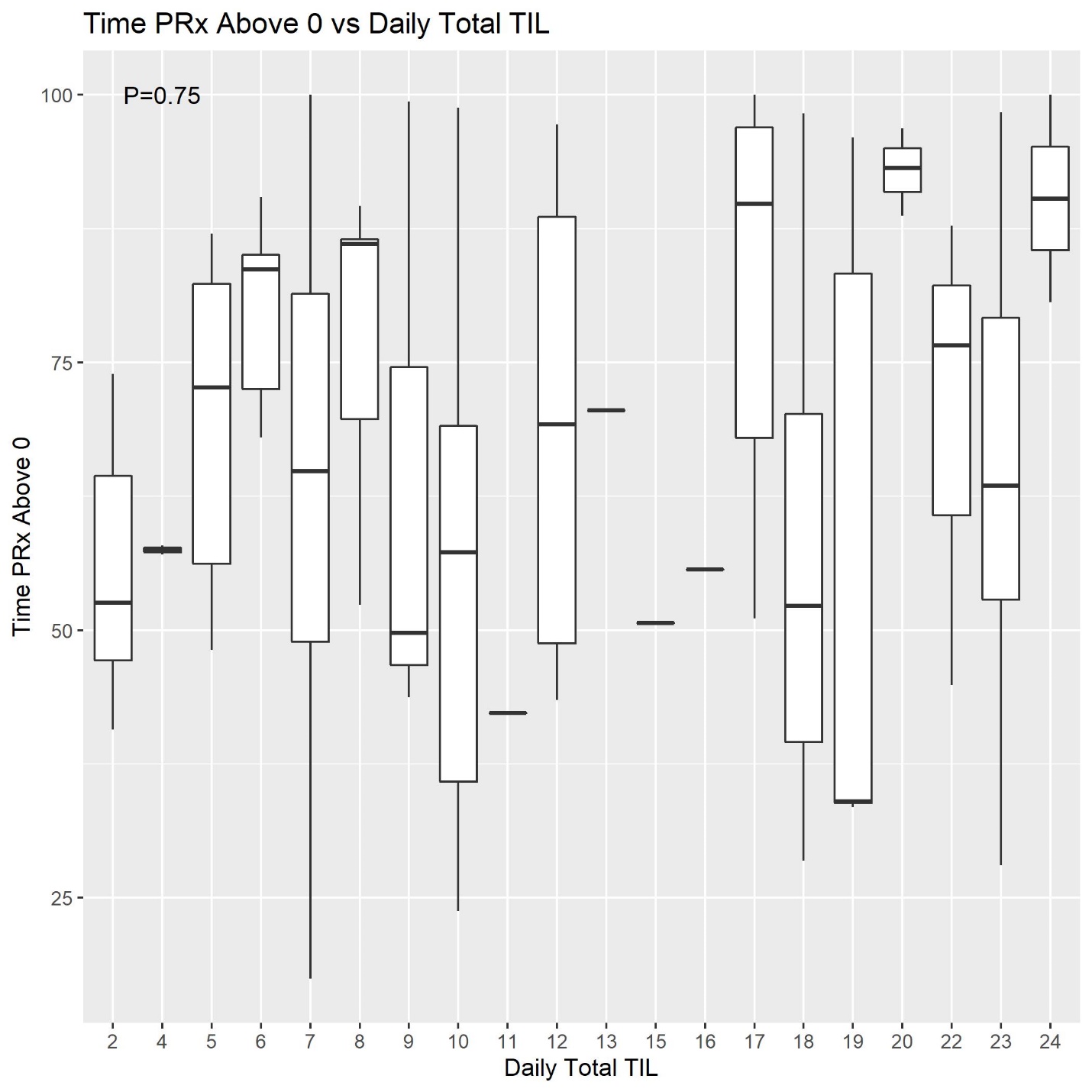

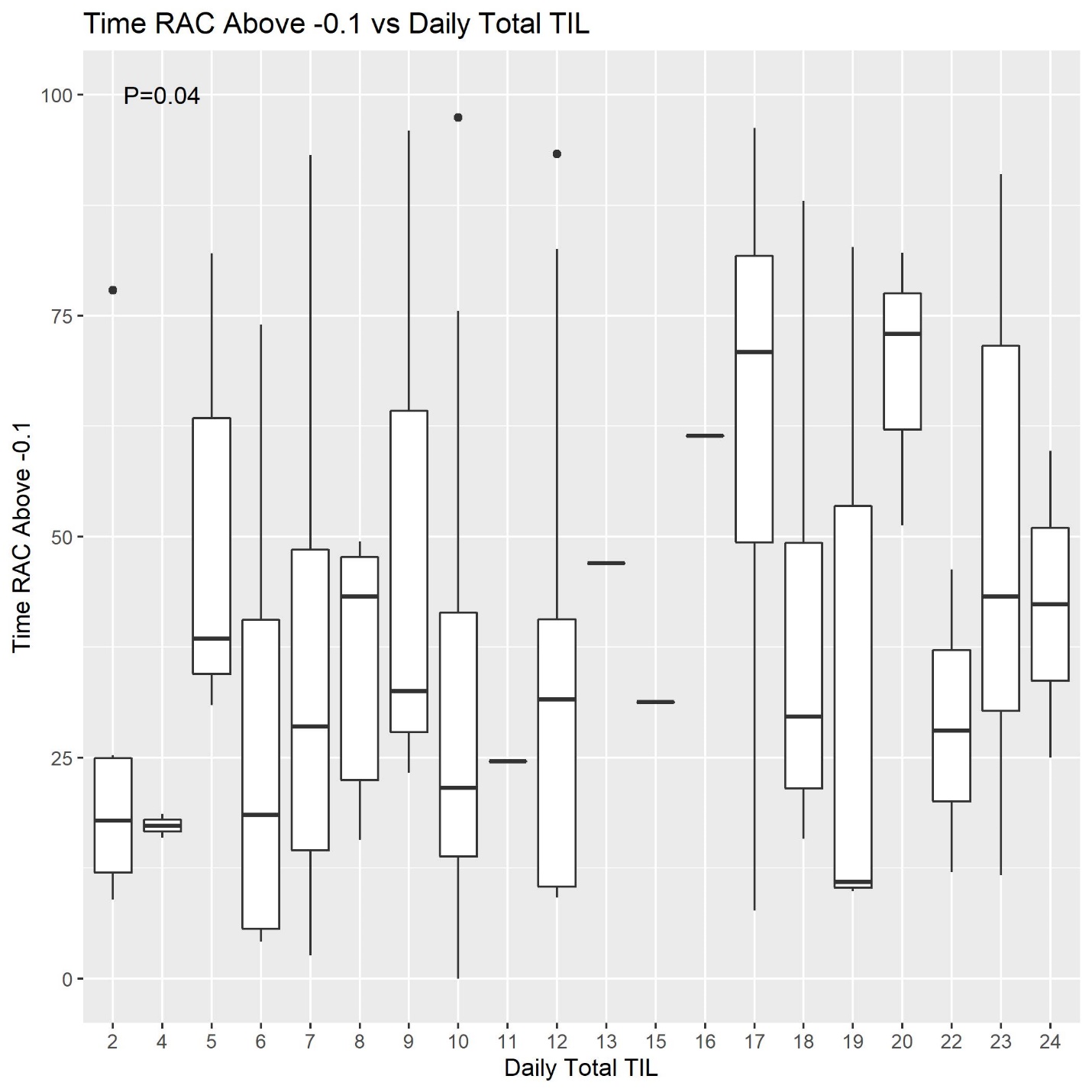

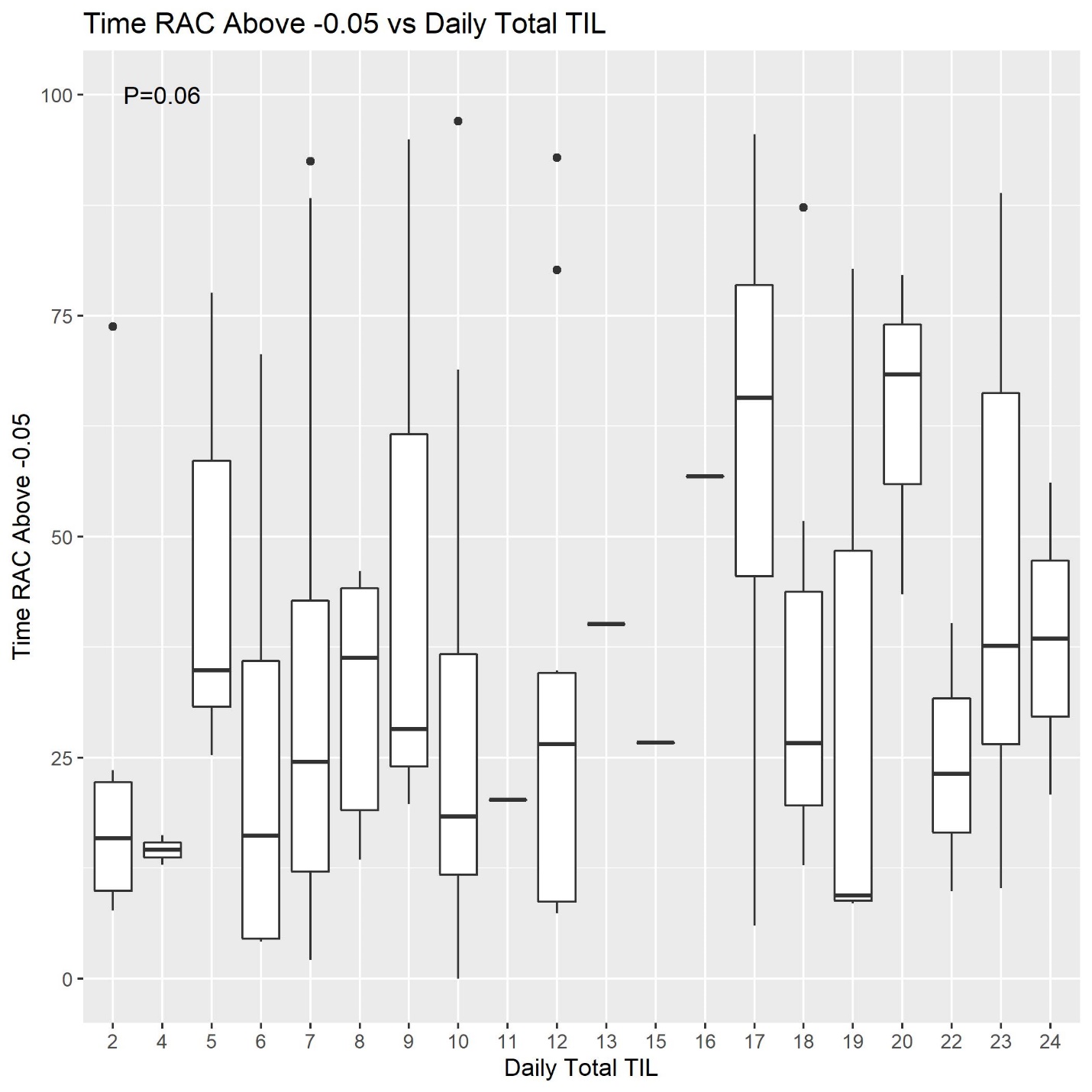
*
